# The impact of obesity on pregnancy outcomes in women with type 1 and type 2 diabetes across the NSW population: A retrospective cohort study

**DOI:** 10.1111/dme.70177

**Published:** 2025-12-03

**Authors:** Xavier Rickard, Matilda S. G. Longfield, Jackson Zhou, Ibinabo Ibiebele, Tessa Weir, Deborah Randall, Siranda Torvaldsen, Felicity Gallimore, Jonathan Morris, Tanya Nippita, Sarah J. Glastras

**Affiliations:** ^1^ Faculty of Medicine and Health University of Sydney Sydney New South Wales Australia; ^2^ The Kolling Institute of Medical Research Northern Sydney Local Health District Sydney New South Wales Australia; ^3^ Department of Diabetes, Endocrinology and Metabolism Royal North Shore Hospital St Leonards New South Wales Australia; ^4^ School of Mathematics and Statistics University of Sydney Sydney New South Wales Australia; ^5^ Reproduction and Perinatal Centre — Northern Precinct, Faculty of Medicine and Health The University of Sydney Sydney New South Wales Australia; ^6^ Department of Diabetes and Endocrinology Nepean‐Blue Mountains Hospital Penrith New South Wales Australia; ^7^ Clinical Excellence Commission, New South Wales Ministry of Health St Leonards New South Wales Australia; ^8^ Department of Obstetrics and Gynaecology Royal North Shore Hospital St Leonards New South Wales Australia

**Keywords:** body mass index, Hyperglycaemia, modelling, obstetrics, Peripartum

## Abstract

**Aims:**

To examine differences in pregnancy outcomes between women with type 1 (T1D) and type 2 diabetes (T2D) and assess the impact of concurrent obesity on adverse perinatal outcomes.

**Materials and Methods:**

We retrospectively analysed singleton births of nulliparous mothers with T1D and T2D from 2016 to 2020, in New South Wales, Australia. The incidence of perinatal outcomes was compared between diabetes types. Logistic regression explored the impact of BMI and diabetes type on these outcomes, adjusting for relevant maternal characteristics.

**Results:**

In total, 568 women with T1D and 910 women with T2D were included. Women with T2D were older, had higher BMI, increased incidence of pre‐existing hypertension and smoking, and higher rates of socioeconomic disadvantage (*p* < 0.01). After stepwise adjustment for maternal covariates, women with T1D had higher odds of preeclampsia, caesarean section, maternal length of stay (LOS) >10 days, preterm birth, large‐for‐gestational age (LGA) neonates, neonatal resuscitation, neonatal hypoglycaemia, NICU admission, neonatal LOS >10 days and stillbirth (aOR >1, *p* < 0.05). Obesity conferred an increased odds of adverse outcomes in women with T2D but not T1D; these included gestational hypertension (aOR = 5.88, CI:1.38–25.15, *p* = 0.02), postpartum haemorrhage (aOR = 1.95, CI:1.18–3.23, *p* < 0.01), caesarean section (aOR = 2.29, CI:1.59–3.30, *p* < 0.01), LGA (aOR = 1.82, CI:1.13–2.93, *p* = 0.01), neonatal hypoglycaemia (aOR = 1.53, CI:1.02–2.30, *p* = 0.04) and neonatal resuscitation (aOR = 1.81, CI:1.19–2.76, *p* < 0.01).

**Conclusion:**

Women with T1D had higher odds of adverse perinatal outcomes compared to those with T2D. Obesity increased risk in women with T2D, but not T1D, raising the possibility that targeted weight interventions in those with T2D may reduce the risk of adverse perinatal outcomes.

**What's new?**

- Adverse perinatal outcomes are more common in women with T1D compared to those with T2D, likely due to longer diabetes duration and poorer glycaemic control, making rigorous glycaemic management essential.
- For women with T2D, effective weight management prior to pregnancy is vital, as obesity significantly increases the risk of adverse maternal and neonatal pregnancy outcomes.
- SUGARED, an Australian data linkage project supported by an NHMRC ideas grant, has made possible the largest Australian assessment of perinatal outcomes in women with pre‐gestational diabetes.

## 1 INTRODUCTION

Obesity is a global health emergency, and its prevalence is increasing rapidly amongst women of childbearing age.^1^ In Australia, nearly 50% of women entering pregnancy in 2021 had overweight (body mass index [BMI] 25–29.9 kg/m^2^) or obesity (BMI ≥30 kg/m^2^).^2^ Obesity adversely affects maternal and neonatal outcomes, increasing the risk of various complications including preeclampsia, caesarean section, postpartum haemorrhage, large for gestational age (LGA) and stillbirth.^3^, ^4^, ^5^, ^6^, ^7^

Pregestational diabetes, including type 1 (T1D) and type 2 diabetes (T2D), affects 0.5%–2.4% of pregnancies globally, with an increasing trend observed annually.^8^ Pregnant women with T1D typically have worse glycaemic control and longer disease duration, whereas women with T2D are older, have higher rates of obesity, insulin resistance, ethnic diversity and socioeconomic deprivation.^9^, ^10^, ^11^ Despite this, rates of obesity are still high in T1D cohorts. A previous Australian study from 2010 to 2013 examining pregnancy outcomes in T1D found that 66% of women with T1D had overweight or obesity, compared with 45% of women without diabetes.^12^

T1D and T2D are both associated with an increased risk of adverse pregnancy outcomes compared with the general population.^9^, ^10^, ^13^, ^14^, ^15^, ^16^, ^17^ A 2024 systematic review identified 35 studies that compared pregnancy outcomes in women with T1D to those with T2D; of these 5 were conducted in Australia in cohorts that ranged from 53 to 198 total participants.^18^ Compared to women with T2D, women with T1D and their infants have been found to experience higher rates of earlier delivery,^10^, ^19^, ^20^ preterm birth,^9^, ^10^, ^18^, ^21^ LGA and macrosomia,^9^, ^10^, ^16^, ^18^, ^21^, ^22^ neonatal intensive care (NICU) admission,^9^, ^10^, ^15^ neonatal hypoglycaemia,^15^ instrumental delivery,^19^, ^22^ pre‐elcampsia^18^, ^21^, ^23^ and caesarean section.^10^, ^20^, ^22^, ^24^ Resulting in longer durations for stay for mothers and neonates.^22^, ^23^ Most studies have shown no difference in neonatal mortality, though a large‐scale meta‐analysis suggests this may be higher in those with T2D,^18^, ^21^ stillbirth and congenital abnormality between the two groups.^13^, ^14^, ^15^, ^16^, ^17^, ^22^, ^23^, ^24^ However, even with optimal glycaemic control, women with T1D and T2D continue to face high rates of pregnancy‐related complications.^25^ Women with T2D showed an almost three‐fold increased incidence of LGA infants (20.3% vs. 7.7%) compared to women without diabetes, despite good glycaemic control and specialist care.^26^ Similarly, another study showed that real‐time continuous glucose monitoring improved glycaemic control in women with T1D, yet the rate of LGA remained high at 53%.^27^ These outcomes suggest that other factors, such as maternal obesity, significantly impact pregnancy outcomes.^28^

This study had two main objectives. First, we aimed to clarify the differences in pregnancy outcomes between T1D and T2D in a large state‐wide Australian population. We hypothesised that the risk of perinatal outcomes would be more severe in T1D, as indicated by previous research. Second, we aimed to investigate the impact of obesity on adverse perinatal outcomes in women with T1D and T2D. Since obesity and pregestational diabetes are both known to independently increase the risk of pregnancy complications, we expected that obesity in conjunction with T1D or T2D would confer an additive risk. Understanding the interplay between obesity and diabetes is vital to help clinicians create personalised interventions to mitigate risks.

## 2 MATERIALS AND METHODS

This study is reported according to the Strengthening the Reporting of Observational Studies in Epidemiology guidelines for cohort studies (Table S1). Ethics approval (2020/ETH02737) was obtained from the NSW Population and Health Services Research Ethics Committee.

### 2.1 Study Population and Data Sources

This retrospective cohort study used routinely collected medical and perinatal surveillance data from the Study of Gestational Diabetes and Risk Using Electronic Data (SUGARED).^29^ The dataset includes all pregnant women and their infants born at ≥20 weeks gestation in NSW hospitals from January 1st, 2016, to December 31st 2020. Data were linked from multiple sources, including the NSW Perinatal Data Collection, Admitted Patient Data Collection, Emergency Department Data Collection, Register of Births, Deaths and Marriages, Births and Deaths Registrations, NSW Health Pathology Data, the Obstetrix database and the eMaternity database. The inclusion criteria were infants born at a gestational age between 25 and 42 weeks to nulliparous mothers aged between 17 and 47 years with either T1D or T2D. The exclusion criteria were mothers with gestational diabetes, other forms of diabetes and those with missing BMI data.

### 2.2 Exposure and Outcome Measures

Baseline maternal characteristics recorded included age, preexisting hypertension, diabetes duration, smoking status, Index of Relative Socio‐economic Advantage and Disadvantage (IRSAD), pre‐pregnancy height and weight, BMI, region of birth, antenatal care status and glycaemic history. The IRSAD score summarises the socioeconomic conditions of an area, with lower scores indicating more disadvantage and higher scores indicating more advantage.^30^ Women were categorised into three weight classes based on BMI according to WHO definitions: normal weight (<25 kg/m^2^), overweight (25–29.9 kg/m^2^) and obesity (≥30 kg/m^2^).^31^ Glycaemic history comprised available HbA1c data pre‐pregnancy, <20 weeks of gestation, trimester 1, trimester 2, trimester 3 and post‐pregnancy. In the case of repeat HbA1c results being available at <20 weeks of gestation, the earliest time point in pregnancy was used.

Maternal outcomes recorded in the study included mode of delivery (caesarean section, instrumental delivery, vaginal delivery), preeclampsia, gestational hypertension, postpartum haemorrhage, maternal hospital length of stay (LOS) categorised as >10 or ≤ 10 days and maternal death. Neonatal outcomes recorded included gestational age, preterm birth (<37 weeks gestation), neonatal resuscitation, neonatal hypoglycaemia, birth weight, LGA (birthweight >90th percentile by gestational age and sex), small for gestational age (SGA, birth weight <10th percentile by gestational age and sex),^32^ neonatal hospital LOS categorised as >10 or ≤ 10 days, NICU admission and stillbirth.

### 2.3 Statistical Analysis

All analyses were performed using R Statistical Software (v4.4.0).^33^ Baseline characteristics and pregnancy outcomes were compared between T1D and T2D. Normality was assessed using QQ plots and D'Agostino's K‐squared test. Quantitative variables were compared using Welch's 2‐sample t‐tests or Wilcoxon rank‐sum tests. Qualitative variables were compared using Chi‐squared tests or Fisher's exact tests.

Three logistic regression models were developed for each outcome. The first model included both the T1D and T2D populations to compare the overall effects between T1D and T2D. The second and third models were stratified by diabetes type, resulting in separate models for T1D and T2D. This stratification allowed for a detailed assessment of the effect of obesity within both T1D and T2D. Univariable analysis was performed initially, using maternal age, duration of diabetes, preexisting hypertension, IRSAD score, smoking status, region of maternal birth, BMI (BMI class in stratified models) and diabetes type (excluded in stratified models). Forward and backward stepwise selection using the Akaike Information Criterion (AIC) was used for multivariable model selection. Complete case analysis was conducted for each model, excluding individuals with missing data specifically in the variables included in the model. Internal validation using 10‐fold cross‐validation was performed to ensure the robustness of the models. The variation inflation factor (VIF) was used to assess multicollinearity between the independent variables. All VIF values were less than 1.5, indicating no problematic multicollinearity. Statistical significance was set at *p* < 0.05. Despite being a retrospective cohort study, bias was minimised by using comprehensive population‐level data which reduced both selection bias and information bias through standardised data collection. Random error was reduced by the large sample size.

A sensitivity analysis was conducted to observe the effect of a 1% increase in HbA1c for women with either T1D or T2D separately. This was done to evaluate the effect of glycaemic control on the incidence of adverse outcomes. This analysis was conducted only in the subset of women for which HbA1c data were available at <20 weeks gestation. Univariable and multivariable analysis was conducted as described previously.

## 3 RESULTS

A total of 1478 births in women aged between 17 and 47 years across NSW with either T1D or T2D were included in this study. The number of participants with missing data for each variable is shown in Table S2. The demographic and clinical characteristics of the population by diabetes type are shown in Table 1. Of the total participants, 568 had T1D and 910 had T2D. Women with T1D were younger than those with T2D (mean age 29.0 ± 5.3 vs. 31.5 ± 5.5 years; *p* < 0.01), more likely to be born in Australia or New Zealand (85.4% vs. 57.4%; *p* < 0.01), had higher IRSAD scores (mean 1001.8 ± 75.0 vs. 982.2 ± 77.1; *p* < 0.01), were taller (mean 165.2 ± 6.8 vs. 163.9 ± 7.1 cm; *p* < 0.01) and had a longer duration of diabetes (median 15.1 ± 8.6 vs. 5.6 ± 3.2 years; *p* < 0.01). Women with T2D had higher BMI than those with T1D (median 30.5, IQR 25.9–35.3 vs. 24.9, IQR 22.6–29.3 kg/m^2^; *p* < 0.01), a higher incidence of smoking during pregnancy (10.2% vs. 6.7%; *p* = 0.03) and a higher incidence of preexisting hypertension (10.7% vs. 6.3%; *p* < 0.01).

**TABLE 1:** Baseline characteristics, by diabetes type.

	Type 1, *N* = 568	Type 2, *N* = 910	*p*‐value
Age (years), mean (SD)	29.0 (5.3)	31.5 (5.5)	<0.001^a^
Duration of diabetes (years), median (IQR)	5.9 (0.5, 12.3)	1.1 (0.0, 3.1)	<0.001^b^
Maternal weight (kg), median (IQR)	69.0 (61.0, 80.0)	80.0 (69.0, 96.0)	<0.001^b^
Maternal height (cm), mean (SD)	165.2 (6.8)	163.9 (7.1)	<0.001^b^
BMI (kg/m^2^), median (IQR)	24.9 (22.6, 29.3)	30.5 (25.9, 35.3)	<0.001^b^
BMI category (kg/m^2^), *n* (%)			<0.001^c^
Normal weight (BMI 18.5–24.9)	290 (51.1%)	189 (20.8%)	
Overweight (BMI 25–29.9)	158 (27.8%)	251 (27.6%)	
Obesity (BMI 30+)	120 (21.1%)	470 (51.6%)	
Antenatal care received, *n* (%)	564 (99.3%)	904 (99.3%)	>0.999^d^
Smoking during pregnancy, *n* (%)	38 (6.7%)	93 (10.2%)	0.026^c^
Preexisting hypertension, *n* (%)	36 (6.3%)	97 (10.7%)	0.006^c^
IRSAD score, mean (SD)	1001.8 (75.0)	982.2 (77.1)	<0.001^a^
Missing (%)	11 (1.9%)	21 (2.3%)	
IRSAD quintile, *n* (%)			<0.001^c^
Most disadvantaged quintile	106 (19.0%)	237 (26.7%)	
2	132 (23.7%)	235 (26.4%)	
3	121 (21.7%)	166 (18.7%)	
4	106 (19.0%)	162 (18.2%)	
Most advantaged quintile	92 (16.5%)	89 (10.0%)	
Missing (%)	11 (1.9%)	21 (2.3%)	
Region of maternal birth, *n* (%)			<0.001^c^
Australia or New Zealand	484 (85.4%)	520 (57.4%)	
Asia	40 (7.1%)	296 (32.7%)	
Europe	29 (5.1%)	45 (5.0%)	
Oceania	<5^e^	23 (2.5%)	
Americas	9 (1.6%)	14 (1.5%)	
Africa	<5^e^	8 (0.9%)	
Missing	1 (0.2%)	4 (0.4%)	
Glycaemic control (HbA1c mmol/mol[%])			
Pre‐pregnancy, median (IQR)	72 (62, 84) [8.7 (7.8, 9.8)]	71 (61, 89) [8.6 (7.7, 10.4)]	0.883^a^
Missing (%)	538 (95%)	905 (99%)	
<20 weeks gestation, median (IQR)	52 (44, 62) [6.9 (6.2, 7.8)]	42 (33, 55) [6.0 (5.2, 7.2)]	<0.001^b^
Missing (%)	464 (81.7%)	776 (85.3%)	
Trimester 1, mean (SD)	57 (18) [7.3 (1.6)]	50 (17) [6.6 (1.6)]	0.006^a^
Missing (%)	490 (86%)	819 (90%)	
Trimester 2, mean (SD)	45 (11) [6.3 (1.0)]	40 (12) [5.8 (1.1)]	0.011^a^
Missing (%)	511 (90%)	847 (93%)	
Trimester 3, mean (SD)	48 (8) [6.4 (0.9)]	44 (12) [6.1 (1.2)]	0.185^a^
Missing (%)	514 (90%)	876 (96%)	
Post‐pregnancy, mean (SD)	60 (22) [8.2 (1.9)]	72 (38) [8.7 (3.2)]	0.689^a^
Missing (%)	543 (96%)	899 (99%)	

*Note*: HbA1c values are shown as mmol/mol (SD) [% (SD%)] for means or mmol/mol (Q1, Q3) [% (Q1, Q3)] for medians; conversion from % used: mmol/mol = 10.93 × % − 23.5, with SDs and IQRs scaled proportionally.

Abbreviations: BMI, body mass index; IQR, interquartile range; IRSAD, index of relative socio‐economic advantage and disadvantage; SD, standard deviation.

^a^ Welch Two Sample *t*‐test.

^b^ Wilcoxon rank sum test.

^c^ Pearson's Chi‐squared test.

^d^ Fisher's exact test.

^e^ Cell counts less than 5 have been suppressed to maintain confidentiality and comply with ethical guidelines.

### 3.1 Maternal outcomes

The differences in adverse perinatal outcomes between the diabetes groups are shown in Table 2. Compared to women with T2D, women with T1D had significantly higher rates of preeclampsia (9.5% vs. 5.8%; *p* = 0.01), postpartum haemorrhage (24.0% vs. 17.7%; *p* < 0.01), caesarean section (69.2% vs. 56.7%; *p* < 0.01) and hospital LOS >10 days (22.2% vs. 8.8%; *p* < 0.01). Compared to women with T1D, women with T2D had significantly higher rates of normal vaginal delivery (28.7% vs. 15.5%; *p* < 0.01). The univariable and multivariable odds ratios of adverse pregnancy outcomes in women with T1D compared to T2D are shown in Table 3. After adjusting for maternal covariates, compared to women with T2D, women with T1D had increased odds of developing preeclampsia (aOR 1.63, CI 1.02–2.60; *p* = 0.04), undergoing a caesarean section (aOR 2.30, CI 1.75–3.02; *p* < 0.01) and having a hospital LOS >10 days (aOR 2.45, CI 1.71–3.53; *p* < 0.01). The maternal characteristics included in adjusted models are included in Table S3. A longer duration of diabetes and preexisting hypertension were both significantly associated with increased odds of preeclampsia and maternal LOS >10 days. The risks of postpartum haemorrhage and gestational hypertension were not significantly different between T1D and T2D.

**TABLE 2:** Pregnancy outcomes, by diabetes type.

	Type 1, *N* = 568^a^	Type 2, *N* = 910^a^	*p*‐value
Preeclampsia	54 (9.5%)	53 (5.8%)	0.011^c^
Gestational hypertension	44 (7.7%)	53 (5.8%)	0.179^c^
Postpartum haemorrhage	136 (24.0%)	161 (17.7%)	0.004^c^
Normal vaginal delivery	88 (15.5%)	261 (28.7%)	<0.001^c^
Instrumental delivery	87 (15.3%)	133 (14.6%)	0.769^c^
Caesarean section	393 (69.2%)	516 (56.7%)	<0.001^c^
Maternal hospital LOS (days)	5.0 (4.0, 8.0)	4.0 (3.0, 5.0)	<0.001^d^
Maternal hospital LOS >10 days	123 (22.2%)	78 (8.8%)	<0.001^c^
Maternal death	0 (0.0%)	0 (0.0%)	>0.999^b^
Gestational age (weeks)	37.0 (36.0, 38.0)	38.0 (37.0, 39.0)	<0.001^d^
Preterm birth	217 (38.9%)	125 (13.8%)	<0.001^c^
Birth weight (kg)	3.5 (3.0, 3.8)	3.2 (2.9, 3.6)	<0.001^d^
SGA	21 (3.8%)	90 (10.0%)	<0.001^c^
LGA	304 (54.5%)	168 (18.6%)	<0.001^c^
NICU admission	146 (64.0%)	126 (36.1%)	<0.001^c^
Neonatal resuscitation	208 (37.3%)	253 (28.0%)	<0.001^c^
Neonatal hypoglycaemia	347 (62.6%)	273 (30.8%)	<0.001^c^
Neonatal hospital LOS (days)	5.0 (4.0, 8.0)	4.0 (3.0, 5.0)	<0.001^d^
Neonatal hospital LOS >10 days	123 (22.2%)	78 (8.8%)	<0.001^c^
Stillbirth	10 (1.8%)	7 (0.8%)	0.137^c^

Abbreviations: IQR, interquartile range; LGA, large for gestational age; LOS, length of stay; NICU, neonatal intensive care; SGA, small for gestational age.

^a^ *n* (%), Median (IQR).

^b^ Fisher's exact test.

^c^ Pearson's Chi‐squared test.

^d^ Wilcoxon rank sum test.

**TABLE 3:** Odds ratios of adverse pregnancy outcomes in women with type 1 diabetes compared to type 2 diabetes.

Outcome	Univariable			Multivariable		
	OR (95% CI)	*p*‐value	*N*	OR (95% CI)	*p*‐value	*N*
Preeclampsia	1.70 (1.14–2.52)	<0.01	1478	1.63 (1.02–2.60)	0.04	1441
Gestational hypertension	1.36 (0.90–2.06)	0.15	1478	—	—	1441
Postpartum haemorrhage	1.47 (1.14–1.90)	<0.01	1476	1.33 (0.98–1.81)	0.07	1439
Caesarean section	1.71 (1.37–2.14)	<0.01	1478	2.30 (1.75–3.02)	<0.01	1441
Maternal hospital LOS >10 days	2.96 (2.18–4.03)	<0.01	1439	2.45 (1.71–3.53)	<0.01	1402
Preterm birth	3.96 (3.07–5.11)	<0.01	1461	3.67 (2.72–4.96)	<0.01	1424
SGA	0.35 (0.22–0.58)	<0.01	1461	0.38 (0.22–0.65)	<0.01	1424
LGA	5.24 (4.13–6.63)	<0.01	1461	5.14 (3.88–6.80)	<0.01	1424
Neonatal resuscitation	1.53 (1.22–1.91)	<0.01	1461	1.41 (1.07–1.86)	0.02	1424
Neonatal hypoglycaemia	3.76 (3.01–4.71)	<0.01	1440	4.21 (3.32–5.34)	<0.01	1403
NICU admission	3.15 (2.22–4.46)	<0.01	577	3.42 (2.23–5.24)	<0.01	560
Neonatal hospital LOS >10days	2.96 (2.17–4.02)	<0.01	1440	2.45 (1.71–3.53)	<0.01	1403
Stillbirth	2.31 (0.87–6.11)	0.09	1478	2.85 (1.06–7.71)	0.04	1441

*Note*: Results are presented as odds ratios (OR) with 95% confidence intervals (CI) and *p*‐values. If no OR is reported within the multivariable analysis, then diabetes type was excluded during the stepwise multivariable model selection based on Akaike Information Criterion (AIC). See Table S3 for the full models with the included covariates.

Abbreviations: LGA, large for gestational age; LOS, length of stay; *N*, denotes the number participants included in the complete case analysis for each model; NICU, neonatal intensive care; SGA, small for gestational age.

Univariable analysis of BMI in the entire cohort revealed that a 1 unit increase in BMI was associated with increased odds of gestational hypertension and caesarean section, even after stepwise adjustment for maternal covariates (aOR 1.04, CI 1.01–1.06; *p* = 0.01 and aOR 1.04, CI 1.02–1.06; *p* < 0.01 respectively, Table S3). A 1 unit increase in BMI was associated with decreased odds of maternal LOS >10 days; however, the association with BMI was excluded by stepwise selection from multivariable models. Conversely, whilst insignificant in univariable analysis, when taken in the context of maternal characteristics such as diabetes type, BMI was a significant variable for increased odds of postpartum haemorrhage (aOR 1.02, CI 1.00–1.04; *p* = 0.04, Table S3). The univariable and multivariable odds ratios of adverse pregnancy outcomes in women with obesity, compared to those with normal weight, in either T1D or T2D, are shown in Table 4. Obesity did not confer an increased risk of any maternal outcome in women with T1D. In women with T2D, obesity increased the risks of developing gestational hypertension (aOR 5.88, CI 1.38–25.15; *p* = 0.02), postpartum haemorrhage (aOR 1.95, CI 1.18–3.23; *p* < 0.01) and caesarean section (aOR 2.29, CI 1.59–3.30; *p* < 0.01).

**TABLE 4:** Odds ratios of adverse pregnancy outcomes in women with obesity compared to normal weight in either type 1 or type 2 diabetes.

Outcome	Type 1						Type 2					
	Univariable			Multivariable			Univariable			Multivariable		
	OR (95% CI)	*p*‐value	*N*	OR (95% CI)	*p*‐value	*N*	OR (95% CI)	*p*‐value	*N*	OR (95% CI)	*p*‐value	*N*
Preeclampsia	1.61 (0.79–3.27)	0.19	568	—	—	556	2.61 (1.08–6.29)	0.03	910	—	—	885
Gestational hypertension	1.11 (0.51–2.42)	0.8	568	—	—	556	7.52 (1.79–31.66)	<0.01	910	5.88 (1.38–25.15)	0.02	885
Postpartum haemorrhage	0.79 (0.47–1.32)	0.37	566	—	—	554	1.79 (1.10–2.90)	0.02	910	1.95 (1.18–3.23)	<0.01	885
Caesarean section	1.19 (0.75–1.91)	0.46	568	—	—	556	2.33 (1.65–3.30)	<0.01	910	2.29 (1.59–3.30)	<0.01	885
Maternal hospital LOS >10 days	1.41 (0.86–2.29)	0.17	553	1.31 (0.79–2.20)	0.3	541	1.87 (0.89–3.94)	0.1	886	1.61 (0.75–3.46)	0.22	861
Preterm birth	1.26 (0.82–1.96)	0.29	558	—	—	546	2.05 (1.14–3.68)	0.02	903	1.81 (0.99–3.31)	0.052	878
SGA	0.65 (0.18–2.37)	0.51	558	—	—	546	0.84 (0.47–1.49)	0.55	903	—	—	878
LGA	1.08 (0.70–1.66)	0.73	558	—	—	546	1.78 (1.11–2.84)	0.02	903	1.82 (1.13–2.93)	0.01	878
Neonatal resuscitation	1.13 (0.73–1.76)	0.57	558	—	—	546	1.98 (1.31–2.99)	<0.01	903	1.81 (1.19–2.76)	<0.01	878
Neonatal hypoglycaemia	0.79 (0.51–1.22)	0.28	554	—	—	542	1.79 (1.21–2.65)	<0.01	886	1.53 (1.02–2.30)	0.04	861
NICU admission	1.26 (0.61–2.60)	0.54	228	—	—	223	1.84 (0.97–3.47)	0.06	349	—	—	337
Neonatal hospital LOS >10 days	1.39 (0.85–2.26)	0.18	554	1.29 (0.77–2.16)	0.33	542	1.87 (0.89–3.94)	0.1	886	1.61 (0.75–3.46)	0.22	861
Stillbirth	0.80 (0.16–4.05)	0.79	568	—	—	556	2.43 (0.29–20.39)	0.41	910	—	—	885

*Note*: Results are presented as odds ratios (OR) with 95% confidence intervals (CI) and *p*‐values. If no OR is reported within the multivariable analysis, then BMI category was excluded during the stepwise multivariable model selection based on Akaike Information Criterion (AIC). See Table S5 for the full models with the included covariates.

Abbreviations: LGA, large for gestational age (>90th percentile); LOS, length of stay; *N*, denotes the number participants included in the complete case analysis for each model; NICU, neonatal intensive care; SGA, small for gestational age (<10th percentile).

### 3.2 Neonatal outcomes

Infants born to women with T1D had significantly higher rates of preterm birth (38.9% vs. 13.8%; *p* < 0.01), LGA (54.5% vs. 18.6%; *p* < 0.01), neonatal hypoglycaemia (62.6% vs. 30.8%; *p* < 0.01), NICU admission (64.0% vs. 36.1%; *p* < 0.01), hospital LOS >10 days (22.2% vs. 8.8%; *p* < 0.01) and neonatal resuscitation (37.3% vs. 28.0%; *p* < 0.01) than women with T2D. Infants born to women with T2D had higher rates of SGA (10.0% vs. 3.8%; *p* < 0.01) (Table 2). After adjusting for maternal covariates, infants born to women with T1D had increased odds of preterm birth (aOR 3.67, CI 2.72–4.96; *p* < 0.01), LGA (aOR 5.14, CI 3.88–6.80; *p* < 0.01), neonatal resuscitation (aOR 1.41, CI 1.07–1.86; *p* = 0.02), neonatal hypoglycaemia (aOR 4.21, CI 3.32–5.34; *p* < 0.01), NICU admission (aOR 3.42, CI 2.23–5.24; *p* < 0.01), hospital LOS >10 days (aOR 2.45, CI 1.71–3.53; *p* < 0.01) and stillbirth (aOR 2.85, CI 1.06–7.71; *p* = 0.04) (Table 3).

In this population, an increase in maternal BMI alone was associated with decreased odds of pre‐term birth, neonatal hypoglycaemia and neonatal LOS >10 days (Table S3). After adjustment for maternal covariates, an increase in BMI was associated with increased odds of LGA neonates and neonatal resuscitation (aOR 1.02, CI 1.00–1.04; *p* = 0.01 and aOR 1.02, CI 1.00–1.04; *p* = 0.02, respectively, Table S3).

After stratification of the population based on diabetes type (Table S4), obesity did not confer an increased risk of any neonatal outcome for infants born to women with T1D. In women with T2D, infants born to women with obesity had increased odds of LGA (aOR 1.82, CI 1.13–2.93; *p* = 0.01), neonatal resuscitation (aOR 1.81, CI 1.19–2.76; *p* < 0.01), neonatal hypoglycaemia (aOR 1.53, CI 1.02–2.30; *p* = 0.04) and hospital LOS >10 days (aOR 1.61, CI 0.75–3.46; *p* = 0.22) (Table 4). The full models for each outcome are available in the appendix (Tables S3 and S5).

### 3.3 The role of glycaemic control

There was a high proportion of missingness in available HbA1c data for each perinatal timepoint (Table 1). The highest volume of HbA1c data was available at <20 weeks of gestation (*n* = 238). The median HbA1c (<20 weeks) for T1D and T2D was 52 (44–62) mmol/mol [6.9 (6.2–7.8)%] and 42 (33–55) mmol/mol [6.0(5.2–5.2)%] respectively. Similarly, HbA1c was also significantly higher in those with T1D compared to those with T2D in trimester 1 and trimester 2 (*p* < 0.05). The maternal characteristics and incidence of perinatal outcomes in the subgroups with HbA1c data available are summarised in Tables S6 and S7. In the subset of patients with available HbA1c data, women with T1D had increased odds of caesarean section, preterm birth, LGA, neonatal resuscitation, neonatal hypoglycaemia and NICU admission, compared to those with T2D after correcting for relevant maternal characteristics (Table S8).

Univariate analysis of the subset of patients with available HbA1c data at <20 weeks gestation revealed that a 1% increase in HbA1c significantly increased the odds of maternal LOS >10 days and neonatal LOS >10 days in T1D, and the odds of caesarean section, neonatal hypoglycaemia and NICU admission were increased in T2D (*p* < 0.05, Table 5). Multivariate analysis revealed that HbA1c was not a significant contributor in any model of adverse perinatal outcomes for those with T1D. Conversely, for those with T2D, HbA1c was a significant contributing variable in multivariate models of caesarean sections, maternal LOS >10 days, preterm birth, neonatal hypoglycaemia, NICU admission and neonatal LOS >10 days (*p* < 0.05, Table 5). In this subset, for those with T2D, obesity was no longer a significant contributing variable in multivariate models of adverse perinatal outcomes. The correlation between BMI and HbA1c in this cohort was weak (r = −0.14), suggesting limited collinearity. This indicates that HbA1c, rather than obesity, is the more important predictor of adverse outcomes in T2D. For those with T1D, obesity decreased the odds of postpartum haemorrhage (*p* < 0.05, Table S9).

**TABLE 5:** Odds ratios of adverse pregnancy outcomes per 1% increase in HbA1c in women with type 1 and type 2 diabetes for those with HbA1c recorded at <20 weeks of gestation.

Outcome	Type 1						Type 2					
	Univariate			Multivariate			Univariate			Multivariate		
	OR (95% CI)	*p*‐value	*N*	OR (95% CI)	*p*‐value	*N*	OR (95% CI)	*p*‐value	*N*	OR (95% CI)	*p*‐value	*N*
Preeclampsia	0.89 (0.57–1.40)	0.61	104	—	—	102	1.11 (0.79–1.56)	0.54	134	—	—	131
Gestational hypertension	0.81 (0.42–1.56)	0.52	104	—	—	102	1.05 (0.63–1.74)	0.86	134	—	—	131
Postpartum haemorrhage	0.98 (0.74–1.30)	0.88	103	—	—	101	1.17 (0.93–1.47)	0.18	134	—	—	131
Caesarean section	1.06 (0.78–1.44)	0.71	104	—	—	102	1.45 (1.09–1.92)	<0.01	134	1.45 (1.10–1.92)	<0.01	131
Maternal hospital LOS >10 days	1.38 (1.04–1.85)	0.03	100	—	—	98	1.38 (0.98–1.96)	0.06	128	1.91 (1.11–3.27)	0.02	125
Preterm birth (<37 weeks)	1.17 (0.91–1.52)	0.22	102	—	—	100	1.25 (0.91–1.70)	0.16	131	1.53 (1.05–2.22)	0.02	128
SGA	0.82 (0.37–1.80)	0.61	102	—	—	100	0.57 (0.27–1.16)	0.12	131	0.58 (0.28–1.18)	0.13	128
LGA	1.00 (0.78–1.29)	0.97	102	—	—	100	0.97 (0.72–1.31)	0.85	131	—	—	128
Neonatal resuscitation	1.22 (0.94–1.59)	0.14	102	—	—	100	1.20 (0.95–1.51)	0.12	131	1.22 (0.96–1.55)	0.1	128
Neonatal hypoglycaemia	1.36 (0.96–1.93)	0.08	101	1.34 (0.95–1.89)	0.09	99	1.37 (1.06–1.76)	0.01	128	1.37 (1.07–1.77)	0.01	125
NICU admission	1.47 (1.01–2.16)	0.04	74	1.32 (0.89–1.94)	0.15	72	1.57 (1.16–2.11)	<0.01	107	1.67 (1.20–2.32)	<0.01	104
Neonatal hospital LOS >10 days	1.39 (1.04–1.85)	0.03	101	—	—	99	1.38 (0.98–1.96)	0.06	128	1.91 (1.11–3.27)	0.02	125
Stillbirth	1.08 (0.47–2.51)	0.85	104	—	—	102	0.71 (0.25–2.02)	0.52	134	—	—	131

*Note*: Results are presented as odds ratios (OR) with 95% confidence intervals (CI), and *p*‐values. If no OR is reported within the multivariate analysis, then HbA1c was excluded during the stepwise multivariate model selection based on Akaike Information Criterion (AIC). See supporting information for full models.

Abbreviations: LGA, large for gestational age (>90th percentile); LOS, length of stay; *N*, denotes the number participants included in the complete case analysis for each model; NICU, neonatal intensive care; SGA, small for gestational age (<10th percentile).

## 4 DISCUSSION

This retrospective study found that the type of diabetes had a more significant influence on the occurrence of pregnancy complications than obesity. We found that women with T1D were younger, more likely to be born in Australia or New Zealand, had lower deprivation scores and a longer diabetes duration than women with T2D, and they had the highest odds of experiencing adverse outcomes even after adjustment for maternal covariates. Women with T2D had higher rates of overweight/obesity, smoking, preexisting hypertension, and more diverse maternal regions of birth. These findings align with previous literature.^9^, ^10^ Obesity did not significantly increase adverse outcomes in women with T1D, whereas obesity conferred increased odds of gestational hypertension, postpartum haemorrhage and caesarean section in women with T2D, with higher odds of neonatal hypoglycaemia, LGA, neonatal resuscitation and extended hospital LOS in the neonates observed. The impact of overweight and obesity on adverse perinatal outcomes for women with T2D has been previously established,^26^ yet the lack of effect of obesity in T1D is unexpected,^28^ and may suggest that factors such as earlier disease onset, increased levels of hyperglycaemia and other factors of glycaemic variability are more influential than body weight on adverse perinatal risk in T1D.

### 4.1 The impact of diabetes type on perinatal outcomes

Our study found high rates of preeclampsia in women with T1D (9.5%) and T2D (5.8%) compared to the 2–8% rate reported in the general population.^34^ Preeclampsia has been associated with high levels of morbidity and mortality in mothers and their neonates.^35^ Notably, the incidence of preeclampsia was higher in women with T1D than T2D, even after adjusting for maternal covariates, despite the higher rates of preexisting hypertension in the T2D cohort. The increased rates of preeclampsia in women with pre‐existing diabetes are well known,^18^ though some previous studies have reported no difference in the incidence of preeclampsia between T1D and T2D.^15^, ^16^, ^17^, ^24^ The difference observed in our study could be due to longer disease duration and suboptimal glycaemic control in those with T1D, leading to microvascular damage and microalbuminuria, compromising placental circulation and increasing preeclampsia risk.^36^ Based on national clinical guidelines, most participants would have been prescribed prophylactic acetylsalicylic acid,^25^ though this data was not explicitly available. Acetylsalicylic acid works by reducing vasoconstriction and acute inflammation, thus reducing the likelihood of preeclampsia.^37^ Despite this, rates of preeclampsia remained high, suggesting that risk may be innate to pre‐pregnancy diabetes irrespective of preventative intervention, and may relate to microvascular damage associated with prolonged periods of hyperglycaemia.^37^ The longer duration of diabetes and poorer glycaemic control in the women with T1D versus T2D, may confer increased risk of pre‐existing microvascular damage increasing the risk of preeclampsia. Furthermore, obstetric‐indicated preterm delivery has been associated with the higher incidence of preeclampsia and macrosomia,^38^ This suggests a high likelihood of co‐occurrence for these adverse outcomes. Consistent with previous studies, our research found a significantly increased incidence of preterm birth in mothers with T1D (38.9%) compared with T2D (13.8%),^9^, ^10^, ^18^, ^21^ with nearly four‐fold increased odds after adjusting for maternal covariates. In women with T1D, spontaneous preterm delivery has also been linked to poor glycaemic control,^39^ which is likely to have been worse overall in the mothers with T1D, compared to T2D, based on the subset of patients for which we had access to HbA1c data.

Neonatal birth weight was significantly higher in neonates of T1D versus T2D mothers (3.5(3.0–3.8)kg vs. 3.2(2.9–3.6)kg, *p* < 0.001). The five‐fold increased risk of LGA in women with T1D versus T2D after adjustment, concordant with previous literature,^9^, ^10^, ^16^, ^18^, ^21^, ^22^ may be explained by increased rates of hyperglycaemia. Poorer glycaemic control leads to elevated maternal glucose levels, which in turn stimulates increased foetal insulin production, promoting growth and fat deposition.^40^ Feig et al. (2022) found that chronic hypertension, nephropathy and metformin use in women with T2D increased the risk of SGA by 25%.^41^ The findings of our study concur with previous research.^9^ Women with T1D had an almost 3‐fold lower odds of SGA neonates than women with T2D, yet the overall incidence of SGA in both groups was low (3.8 and 10.0%, respectively), In our study, subset analysis revealed higher HbA1c levels in women with T1D and higher levels of pre‐existing hypertension in women with T2D. These factors in combination may explain the disparity in neonatal size observed between the neonates of women with T1D and T2D.

Alongside increased rates of LGA, our study found a markedly increased incidence of caesarean sections,^10^, ^16^, ^20^, ^22^, ^24^ maternal LOS >10 days,^9^, ^10^, ^16^, ^18^, ^21^, ^22^, ^23^ in T1D versus T2D. Women with T1D were more likely to experience post‐partum haemorrhage than those with T2D. The rates of caesarean section were significantly higher in our study than the Australian population rate of 38% in 2021.^2^ Due to the reporting of this retrospective data, we were unable to delineate between emergent and scheduled caesarean sections. However, as all included women were nulliparous, all were primary caesarean sections. We observed a higher incidence in women with T1D (69.2%) compared to T2D (56.7%), with the T1D group having two‐fold increased odds even after adjustment. Previous studies corroborate that the incidence of caesarean section is higher in T1D compared to T2D,^10^, ^20^, ^22^, ^24^ though other studies have found no difference.^15^, ^16^, ^17^ High rates of caesarean delivery in patients with T1D could be explained by the high rates of LGA infants in this group.^10^ Vaginal delivery of LGA neonates carries an increased risk of birth trauma, such as shoulder dystocia,^42^ and uterine atony and increased bleeding, often steering obstetric decision‐making towards primary caesarean section.^43^ Collectively, higher rates of caesarean section and delivery complications in T1D mothers could be contributing to their increased likelihood of requiring longer LOS.^44^ Our study found that women with T1D had nearly a 2.5‐fold increased odds of a maternal LOS exceeding 10 days compared to those with T2D. a finding that is concurrent with previous literature.^22^, ^23^

NICU admission rates and neonatal hospital LOS were notably higher for T1D neonates compared to T2D, likely reflecting elevated rates of neonatal hypoglycaemia, neonatal resuscitation, LGA and preterm birth. However, NICU admission data was the single variable with the highest level of missing data, with data available for 39.3% of patients with T1D and 37.0% with T2,D; therefore these results should be interpreted with caution. It is well known that preexisting diabetes is a risk factor for neonatal hypoglycaemia. Similar to previous studies,^15^, ^45^ our analysis showed that infants born to mothers with T1D had 2.56 times higher adjusted odds of neonatal hypoglycaemia than those with T2D. Literature suggests that higher hyperglycaemia rates in T1D lead to continuous placental transport of glucose from the mother to the foetus, resulting in neonatal hyperinsulinaemia and subsequent lower postnatal glucose levels.^46^ Furthermore, our study found that infants born to mothers with T1D had higher rates of neonatal resuscitation being required, a trend that persisted after adjustment. This is likely due to increased rates of respiratory distress syndrome (RDS), which requires resuscitation,^47^, ^48^ though RDS was not recorded in this study. Few details were available in this retrospective dataset to indicate the potential severity included in the definition of neonatal resuscitation. As neonatal resuscitation was reported in 37% of the overall cohort, we assume a broad spectrum of intervention from mild assistive support to complete cardiopulmonary resuscitation.

No significant differences were observed in the incidence of stillbirth or instrumental delivery between mothers with T1D or T2D, and there was no maternal mortality. The incidence of stillbirth was low (1.2%), yet T1D was associated with higher odds of stillbirth, after adjustment for relevant maternal covariates. Due to the low incidence, this finding should be interpreted with caution, as this is contra to findings obtained in the meta‐analysis conducted by Drabløs et al.^18^

### 4.2 The influence of BMI on perinatal outcomes

In this cohort, 79.2% of women with T2D had overweight or obesity, compared to 48.9% of women with T1D, the latter similar to the background general population rates of overweight and obesity (45.7%, NSW Ministry of Health).^49^ Whilst high, these findings are concordant with previous studies^9^, ^10^, ^12^ In the multivariate models of adverse perinatal outcomes, BMI only contributed to the risk of gestational hypertension, post‐partum haemorrhage, caesarean section and LGA neonates. Obesity is strongly correlated with gestational hypertension due to its association with insulin resistance, systemic inflammation and oxidative stress.^50^ In the overall population we observed that a 1 unit increase in BMI increased the odds of gestational hypertension by 4%. In women with T2D, obesity increased the odds of gestational hypertension six‐fold compared to those with normal weight. This same association was not observed in those with T1D. This may be attributed to higher levels of obesity, and therefore adiposity in those with T2D compared to those with T1D. Contrary to expectations, obesity did not influence preeclampsia risk in either T1D or T2D, despite a systematic review indicating that the unadjusted risk of preeclampsia doubles for every 5 to 7 unit increase in BMI.^51^ This discrepancy may be due to our adjustment for confounders including preexisting hypertension, which reduced the observed effect of obesity on preeclampsia. This suggests that preexisting hypertension was a highly influential risk factor on preeclampsia risk irrespective of BMI.

Obesity contributes to larger foetal size, prolonged labour and a higher likelihood of labour induction, all of which are associated with an increased risk of caesarean delivery.^52^, ^53^ This suggests that the associations identified between BMI and LGA neonates, as previously stated, may be influencing the observed patterns in both caesarean sections and postpartum haemorrhage. A 1‐unit increase in BMI was associated with a 4% increased likelihood of caesarean section, and a 2% increased likelihood of postpartum haemorrhage and LGA in the overall population, potentially driven by the 2‐fold association between obesity and these outcomes in those with T2D. The association between BMI and adverse pregnancy outcomes was not observed in women with T1D. Obesity class is independently associated with LGA incidence,^54^ caesarean section^7^ and postpartum haemorrhage,^55^, ^56^ regardless of diabetes status and despite guidelines for women with obesity to preferably undergo vaginal delivery.^4^

Higher birth weight is known to be associated with higher rates of neonatal hypoglycaemia, shoulder dystocia and NICU admission, as well as increased risk of obesity, T2D and chronic diseases later in life.^57^, ^58^ In women with T2D, obesity was additionally associated with an increased odds of neonatal resuscitation and hypoglycaemia, confirming previous findings.^59^ However, another study found that neonatal hypoglycaemia was more frequent in infants of mothers with T1D than T2D, but was not influenced by maternal weight.^44^ Further investigation is required to explore additional cofactors not measured in this cohort, such as diabetic medication use and weight gain during pregnancy.

### 4.3 The effect of glycaemic control on perinatal outcomes

Many national and international guidelines recommend a target HbA1c of 6.5% in trimester 1, and 6.0% in trimesters 2 and 3.^25^ In our sub‐analysis of participants with HbA1c data available, just 36.5% of women with T1D reached target HbA1c <6.5% within the first 20 weeks of gestation, whereas 61.2% of T2D achieved this target, similar to or better than reports in previous studies.^9^, ^10^ Incorporating HbA1c results from <20 weeks gestation into models of adverse perinatal outcomes in our sensitivity analysis revealed that glycaemic control is a driving factor of differences observed between outcomes from women with T1D versus T2D. It is well established that even a modest reduction in HbA1c significantly reduces the incidence of LGA neonates, neonatal hypoglycaemia, preterm birth and NICU admission.^59^, ^60^ Despite this knowledge, our sensitivity analysis did not reveal an association between increased HbA1c and odds of LGA neonates in either T1D or T2D, despite comparable rates of LGA in the subset with known HbA1c compared to the overall cohort. Further exploration is therefore still required to establish the potential mechanisms conferring high rates of LGA irrespective of HbA1c in pregnancy.

## 5 LIMITATIONS

Our study had several limitations. As a retrospective population‐based cohort study, it was subject to missing data. Current clinical recommendations highlight the importance of pre‐pregnancy evaluation of HbA1c and the utility of inter‐pregnancy measurement of HbA1c.^25^ The availability of HbA1c throughout pregnancy was highly variable, and importantly we did not have pre‐pregnancy HbA1c data available. This is notable as higher HbA1c levels, particularly pre‐pregnancy and during the first trimester, have been associated with various pregnancy complications, including LGA, preterm birth and congenital abnormalities.^9^, ^38^, ^44^, ^60^, ^61^, ^62^ We also lacked information on medications, diet, exercise and gestational weight gain, which are associated with metabolic control, hyperglycaemia and pregnancy outcomes.^28^, ^63^, ^64^, ^65^ The impact of maternal ethnicity on perinatal risk was also unable to be explored in this cohort due to limitations in hospital reporting. Ethnicity data was approximated using country of birth instead of specific ethnic categories, an approach with recognised shortcomings.^66^ As such this measure may better represent cultural diversity, as Australia is recognised as having high levels of ethnic diversity.^66^ As such, BMI categories were also not adjusted for ethnicity, which may affect the accuracy of our obesity‐related findings, as different ethnic groups have varying BMI thresholds for health risks.^67^, ^68^

## 6 CONCLUSIONS

These findings highlight the urgent need for personalised antenatal care to address the distinct risk profiles of women with T1D and T2D. Although obesity did not have a significant impact on T1D outcomes in this study, clinicians should be aware that women with T1D experience the most severe adverse outcomes, likely due to longer diabetes duration and poorer glycaemic control, making rigorous glycaemic management essential. In contrast, for women with T2D, effective weight management prior to pregnancy is vital, as obesity significantly increases the risk of adverse maternal and neonatal pregnancy outcomes. Future research should explore these findings in controlled observational clinical trials. This setting would allow for the exploration of additional maternal risk factors on adverse pregnancy outcomes not investigated here, such as pre‐pregnancy metabolic control, more complete HbA1c results, glycaemic variability and gestational weight gain, to further explore their impact on perinatal risk. This would in turn help to refine personalised care strategies and improve pregnancy outcomes.

## FUNDING

This study was supported by the National Health and Medical Research Foundation (Ideas Grant GNT1186572) and in‐kind support from the Department of Diabetes, Metabolism and Endocrinology, Royal North Shore Hospital. Publication will be paid for by the Department of Diabetes, Metabolism and Endocrinology, Royal North Shore Hospital.

## CONFLICT OF INTEREST STATEMENT

The authors have declared that no competing interests exist.

## Supporting information


**Table S1.** STROBE Statement ‐ Checklist of items that should be included in reports of cohort studies.
**Table S2.** Number and percentage of missing observations for each variable.
**Table S3.** Univariable and multivariable logistic regression models of perinatal outcomes in women with type 1 and type 2 diabetes.
**Table S4.** Maternal characteristics of all patients, by diabetes type and obesity status.
**Table S5.** Univariable and multivariable logistic regression models of perinatal outcomes, stratified by type 1 and type 2 diabetes.
**Table S6.** Maternal characteristics of patients with a recorded HbA1c <20 weeks of gestation, by diabetes type.
**Table S7.** Pregnancy outcomes, by diabetes type in the subset of women with HbA1c.
**Table S8.** Odds ratios of adverse pregnancy outcomes in women with type 1 diabetes compared to type 2 diabetes in the subset of women with HbA1c available at <20 weeks of gestation.
**Table S9.** Odds ratios of adverse pregnancy outcomes in women with obesity compared to normal weight in either type 1 or type 2 diabetes in subset of women with HbA1c available at <20 weeks gestation.
