# Supplementary material for: The impact of obesity on pregnancy outcomes in women with type 1 and type 2 diabetes across the NSW population: A retrospective cohort study
Source: Diabet Med. 2025 Dec 3;43(2):e70177. doi: 10.1111/dme.70177 (PMC12857879; doi:10.1111/dme.70177)
Supplement: Supplementary file 1 — Table S1. STROBE Statement ‐ Checklist of items that should be included in reports of cohort studies. Table S2. Number and percentage of missing observations for each variable. Table S3. Univariable and multivariable logistic regression models of perinatal outcomes in women with type 1 and type 2 diabetes. Table S4. Maternal characteristics of all patients, by diabetes type and obesity status. Table S5. Univariable and multivariable logistic regression models of perinatal outcomes, stratified by type 1 and type 2 diabetes. Table S6. Maternal characteristics of patients with a recorded HbA1c <20 weeks of gestation, by diabetes type. Table S7. Pregnancy outcomes, by diabetes type in the subset of women with HbA1c. Table S8. Odds ratios of adverse pregnancy outcomes in women with type 1 diabetes compared to type 2 diabetes in the subset of women with HbA1c available at <20 weeks of gestation. Table S9. Odds ratios of adverse pregnancy outcomes in women with obesity compared to normal weight in either type 1 or type 2 diabetes in subset of women with HbA1c available at <20 weeks gestation. [file DME-43-e70177-s001.docx]

**Supplementary Appendix**

**Table of Contents**

Supplementary Table 1 …….…….…….…….……. 2

Supplementary Table 2 …….…….…….…….……. 5

Supplementary Table 3 …….…….…….…….……. 6

Supplementary Table 4 …….…….…….…….……. 12

Supplementary Table 5 …….…….…….…….……. 14

Supplementary Table 6 …….…….…….…….……. 30

Supplementary Table 7 …….…….…….…….……. 32

Supplementary Table 8 …….…….…….…….……. 33

Supplementary Table 9 …….…….…….…….……. 34

**Supplementary Table 1: STROBE Statement - Checklist of items that should be included in reports of cohort studies**

|  | Item No | Recommendation | Page No |
| --- | --- | --- | --- |
| **Title and abstract** | 1 | (*a*) Indicate the study’s design with a commonly used term in the title or the abstract | 1 |
|  |  | (*b*) Provide in the abstract an informative and balanced summary of what was done and what was found | 2-3 |
| Introduction | | | |
| Background/rationale | 2 | Explain the scientific background and rationale for the investigation being reported | 4 |
| Objectives | 3 | State specific objectives, including any prespecified hypotheses | 5 |
| Methods | | | |
| Study design | 4 | Present key elements of study design early in the paper | 5 |
| Setting | 5 | Describe the setting, locations, and relevant dates, including periods of recruitment, exposure, follow-up, and data collection | 5 |
| Participants | 6 | (*a*) Give the eligibility criteria, and the sources and methods of selection of participants. Describe methods of follow-up | 6 |
|  |  | (*b*) For matched studies, give matching criteria and number of exposed and unexposed |  |
| Variables | 7 | Clearly define all outcomes, exposures, predictors, potential confounders, and effect modifiers. Give diagnostic criteria, if applicable | 6 |
| Data sources/ measurement | 8* | For each variable of interest, give sources of data and details of methods of assessment (measurement). Describe comparability of assessment methods if there is more than one group | 6 |
| Bias | 9 | Describe any efforts to address potential sources of bias | 7 |
| Study size | 10 | Explain how the study size was arrived at | 5, 7 |
| Quantitative variables | 11 | Explain how quantitative variables were handled in the analyses. If applicable, describe which groupings were chosen and why | 6-7 |
| Statistical methods | 12 | (*a*) Describe all statistical methods, including those used to control for confounding | 6-7 |
|  |  | (*b*) Describe any methods used to examine subgroups and interactions | 6-7 |
|  |  | (*c*) Explain how missing data were addressed | 7 |
|  |  | (*d*) If applicable, explain how loss to follow-up was addressed |  |
|  |  | (*e*) Describe any sensitivity analyses |  |
| Results | | |  |
| Participants | 13* | (a) Report numbers of individuals at each stage of study—eg numbers potentially eligible, examined for eligibility, confirmed eligible, included in the study, completing follow-up, and analysed | 7 |
|  |  | (b) Give reasons for non-participation at each stage |  |
|  |  | (c) Consider use of a flow diagram |  |
| Descriptive data | 14* | (a) Give characteristics of study participants (eg demographic, clinical, social) and information on exposures and potential confounders | 7-8 |
|  |  | (b) Indicate number of participants with missing data for each variable of interest | Supplementary table 2 |
|  |  | (c) Summarise follow-up time (eg, average and total amount) |  |
| Outcome data | 15* | Report numbers of outcome events or summary measures over time | 8-9 |
| Main results | 16 | (*a*) Give unadjusted estimates and, if applicable, confounder-adjusted estimates and their precision (eg, 95% confidence interval). Make clear which confounders were adjusted for and why they were included | 8-9 |
|  |  | (*b*) Report category boundaries when continuous variables were categorized | 8-9 |
|  |  | (*c*) If relevant, consider translating estimates of relative risk into absolute risk for a meaningful time period |  |
| Other analyses | 17 | Report other analyses done—eg analyses of subgroups and interactions, and sensitivity analyses | 8-9 |
| **Discussion** |  |  |  |
| Key results | 18 | Summarise key results with reference to study objectives | 9 |
| Limitations | 19 | Discuss limitations of the study, taking into account sources of potential bias or imprecision. Discuss both direction and magnitude of any potential bias | 14 |
| Generalisability | 21 | Discuss the generalisability (external validity) of the study results | 14 |
| **Other information** |  |  |  |
| Funding | 22 | Give the source of funding and the role of the funders for the present study and, if applicable, for the original study on which the present article is based | 14-15 |

*Give information separately for exposed and unexposed groups.

**Note:** An Explanation and Elaboration article discusses each checklist item and gives methodological background and published examples of transparent reporting. The STROBE checklist is best used in conjunction with this article (freely available on the Web sites of PLoS Medicine at http://www.plosmedicine.org/, Annals of Internal Medicine at http://www.annals.org/, and Epidemiology at http://www.epidem.com/). Information on the STROBE Initiative is available at http://www.strobe-statement.org.

**Supplementary Table 2: Number and percentage of missing observations for each variable**

| **Variable** | **Number of Missing Values** | **Percentage Missing** |
| --- | --- | --- |
| NICU admission | 901 | 61.0 |
| Maternal hospital length of stay (days) | 39 | 2.64 |
| Maternal hospital length of stay > 10 days | 39 | 2.64 |
| Neonatal hypoglycaemia | 38 | 2.57 |
| Neonatal hospital length of stay (days) | 38 | 2.57 |
| Neonatal hospital length of stay > 10 days | 38 | 2.57 |
| IRSAD score | 32 | 2.17 |
| IRSAD quintile | 32 | 2.17 |
| Preterm birth | 17 | 1.15 |
| Birth weight (kg) | 17 | 1.15 |
| SGA | 17 | 1.15 |
| LGA | 17 | 1.15 |
| Neonatal resuscitation | 17 | 1.15 |
| Maternal death | 6 | 0.406 |
| Region of maternal birth | 5 | 0.338 |
| Postpartum haemorrhage | 2 | 0.135 |
| Diabetes type | 0 | 0 |
| Age (years) | 0 | 0 |
| Duration of diabetes (years) | 0 | 0 |
| Maternal weight (kg) | 0 | 0 |
| Maternal height (cm) | 0 | 0 |
| BMI (kg/m²) | 0 | 0 |
| BMI category (kg/m²) | 0 | 0 |
| Antenatal care received | 0 | 0 |
| Smoking during pregnancy | 0 | 0 |
| Pre-existing hypertension | 0 | 0 |
| Preeclampsia | 0 | 0 |
| Gestational hypertension | 0 | 0 |
| Normal vaginal delivery | 0 | 0 |
| Instrumental delivery | 0 | 0 |
| Caesarean section | 0 | 0 |
| Gestational age (weeks) | 0 | 0 |
| Stillbirth | 0 | 0 |

**Supplementary Table 3: Univariable and multivariable logistic regression models of perinatal outcomes in women with type 1 and type 2 diabetes**

|  | | | **Univariable** | | | **Multivariable** | | | | |  |
| --- | --- | --- | --- | --- | --- | --- | --- | --- | --- | --- | --- |
| **Outcome** | **Predictor** | **OR (95% CI)** | | **p-value** | **N** | | **OR (95% CI)** | **p-value** | **N** | **AUC** | |
| **Preeclampsia** | Diabetes type |  | |  | 1478 | |  |  | 1441 | 0.71 | |
|  | Type 2 | REF | | REF |  | | REF | REF |  |  | |
|  | Type 1 | 1.70 (1.14-2.52) | | <0.01 |  | | 1.63 (1.02-2.60) | 0.04 |  |  | |
|  | BMI | 1.01 (0.99-1.04) | | 0.37 | 1478 | | -- | -- |  |  | |
|  | Duration of diabetes | 1.07 (1.03-1.11) | | <0.01 | 1478 | | 1.05 (1.01-1.10) | 0.03 |  |  | |
|  | Age | 1.00 (0.97-1.04) | | 0.97 | 1478 | | -- | -- |  |  | |
|  | IRSAD score | 1.00 (1.00-1.00) | | 0.54 | 1446 | | -- | -- |  |  | |
|  | Preexisting hypertension |  | |  | 1478 | |  |  |  |  | |
|  | No | REF | | REF |  | | REF | REF |  |  | |
|  | Yes | 6.31 (4.01-9.94) | | <0.01 |  | | 6.96 (4.35-11.13) | <0.01 |  |  | |
|  | Smoking during pregnancy |  | |  | 1478 | |  |  |  |  | |
|  | No | REF | | REF |  | | -- | -- |  |  | |
|  | Yes | 1.19 (0.62-2.29) | | 0.59 |  | | -- | -- |  |  | |
|  | Region of maternal birth |  | |  | 1473 | |  |  |  |  | |
|  | Australia or New Zealand | REF | | REF |  | | -- | -- |  |  | |
|  | Asia | 0.40 (0.22-0.74) | | <0.01 |  | | -- | -- |  |  | |
|  | Europe | 1.31 (0.61-2.82) | | 0.49 |  | | -- | -- |  |  | |
|  | Americas | 0.00 (0.00-Inf) | | 0.99 |  | | -- | -- |  |  | |
|  | Africa | 0.00 (0.00-Inf) | | 0.99 |  | | -- | -- |  |  | |
|  | Oceania | 0.98 (0.23-4.26) | | 0.98 |  | | -- | -- |  |  | |
| **Gestational hypertension** | Diabetes type |  | |  | 1478 | |  |  | 1441 | 0.63 | |
|  | Type 2 | REF | | REF |  | | -- | -- |  |  | |
|  | Type 1 | 1.36 (0.90-2.06) | | 0.15 |  | | -- | -- |  |  | |
|  | BMI | 1.04 (1.01-1.07) | | <0.01 | 1478 | | 1.04 (1.01-1.06) | 0.01 |  |  | |
|  | Duration of diabetes | 1.00 (0.96-1.05) | | 0.93 | 1478 | | -- | -- |  |  | |
|  | Age | 1.01 (0.97-1.05) | | 0.56 | 1478 | | -- | -- |  |  | |
|  | IRSAD score | 1.00 (1.00-1.00) | | 0.62 | 1446 | | -- | -- |  |  | |
|  | Preexisting hypertension |  | |  | 1478 | |  |  |  |  | |
|  | No | REF | | REF |  | | -- | -- |  |  | |
|  | Yes | 1.32 (0.69-2.54) | | 0.41 |  | | -- | -- |  |  | |
|  | Smoking during pregnancy |  | |  | 1478 | |  |  |  |  | |
|  | No | REF | | REF |  | | REF | REF |  |  | |
|  | Yes | 0.66 (0.28-1.55) | | 0.34 |  | | 0.46 (0.18-1.16) | 0.1 |  |  | |
|  | Region of maternal birth |  | |  | 1473 | |  |  |  |  | |
|  | Australia or New Zealand | REF | | REF |  | | REF | REF |  |  | |
|  | Asia | 0.47 (0.26-0.86) | | 0.01 |  | | 0.50 (0.27-0.92) | 0.03 |  |  | |
|  | Europe | 0.33 (0.08-1.35) | | 0.12 |  | | 0.34 (0.08-1.41) | 0.14 |  |  | |
|  | Americas | 0.53 (0.07-4.01) | | 0.54 |  | | 0.00 (0.00-Inf) | 0.99 |  |  | |
|  | Africa | 1.06 (0.14-8.37) | | 0.95 |  | | 1.04 (0.13-8.20) | 0.97 |  |  | |
|  | Oceania | 0.00 (0.00-Inf) | | 0.98 |  | | 0.00 (0.00-Inf) | 0.98 |  |  | |
| **Postpartum haemorrhage** | Diabetes type |  | |  | 1476 | |  |  | 1439 | 0.59 | |
|  | Type 2 | REF | | REF |  | | REF | REF |  |  | |
|  | Type 1 | 1.47 (1.14-1.90) | | <0.01 |  | | 1.33 (0.98-1.81) | 0.07 |  |  | |
|  | BMI | 1.01 (0.99-1.03) | | 0.26 | 1476 | | 1.02 (1.00-1.04) | 0.04 |  |  | |
|  | Duration of diabetes | 1.05 (1.03-1.08) | | <0.01 | 1476 | | 1.04 (1.01-1.07) | <0.01 |  |  | |
|  | Age | 0.99 (0.97-1.01) | | 0.47 | 1476 | | -- | -- |  |  | |
|  | IRSAD score | 1.00 (1.00-1.00) | | 0.51 | 1444 | | -- | -- |  |  | |
|  | Preexisting hypertension |  | |  | 1476 | |  |  |  |  | |
|  | No | REF | | REF |  | | -- | -- |  |  | |
|  | Yes | 0.82 (0.51-1.30) | | 0.39 |  | | -- | -- |  |  | |
|  | Smoking during pregnancy |  | |  | 1476 | |  |  |  |  | |
|  | No | REF | | REF |  | | -- | -- |  |  | |
|  | Yes | 1.14 (0.74-1.76) | | 0.55 |  | | -- | -- |  |  | |
|  | Region of maternal birth |  | |  | 1471 | |  |  |  |  | |
|  | Australia or New Zealand | REF | | REF |  | | -- | -- |  |  | |
|  | Asia | 0.78 (0.56-1.07) | | 0.12 |  | | -- | -- |  |  | |
|  | Europe | 1.38 (0.81-2.36) | | 0.24 |  | | -- | -- |  |  | |
|  | Americas | 0.78 (0.26-2.33) | | 0.66 |  | | -- | -- |  |  | |
|  | Africa | 0.34 (0.04-2.64) | | 0.3 |  | | -- | -- |  |  | |
|  | Oceania | 0.16 (0.02-1.21) | | 0.08 |  | | -- | -- |  |  | |
| **Caesarean section** | Diabetes type |  | |  | 1478 | |  |  | 1441 | 0.65 | |
|  | Type 2 | REF | | REF |  | | REF | REF |  |  | |
|  | Type 1 | 1.71 (1.37-2.14) | | <0.01 |  | | 2.30 (1.75-3.02) | <0.01 |  |  | |
|  | BMI | 1.02 (1.00-1.04) | | 0.01 | 1478 | | 1.04 (1.02-1.06) | <0.01 |  |  | |
|  | Duration of diabetes | 1.04 (1.02-1.06) | | <0.01 | 1478 | | 1.03 (1.00-1.05) | 0.054 |  |  | |
|  | Age | 1.06 (1.04-1.08) | | <0.01 | 1478 | | 1.08 (1.05-1.10) | <0.01 |  |  | |
|  | IRSAD score | 1.00 (1.00-1.00) | | 0.06 | 1446 | | -- | -- |  |  | |
|  | Preexisting hypertension |  | |  | 1478 | |  |  |  |  | |
|  | No | REF | | REF |  | | REF | REF |  |  | |
|  | Yes | 1.92 (1.28-2.88) | | <0.01 |  | | 1.51 (0.99-2.31) | 0.06 |  |  | |
|  | Smoking during pregnancy |  | |  | 1478 | |  |  |  |  | |
|  | No | REF | | REF |  | | -- | -- |  |  | |
|  | Yes | 0.80 (0.55-1.14) | | 0.22 |  | | -- | -- |  |  | |
|  | Region of maternal birth |  | |  | 1473 | |  |  |  |  | |
|  | Australia or New Zealand | REF | | REF |  | | -- | -- |  |  | |
|  | Asia | 0.95 (0.73-1.22) | | 0.66 |  | | -- | -- |  |  | |
|  | Europe | 1.29 (0.78-2.13) | | 0.32 |  | | -- | -- |  |  | |
|  | Americas | 0.57 (0.25-1.30) | | 0.18 |  | | -- | -- |  |  | |
|  | Africa | 0.62 (0.20-1.94) | | 0.41 |  | | -- | -- |  |  | |
|  | Oceania | 1.03 (0.45-2.38) | | 0.94 |  | | -- | -- |  |  | |
| **Maternal hospital LOS > 10 days** | Diabetes type |  | |  | 1439 | |  |  | 1402 | 0.68 | |
|  | Type 2 | REF | | REF |  | | REF | REF |  |  | |
|  | Type 1 | 2.96 (2.18-4.03) | | <0.01 |  | | 2.45 (1.71-3.53) | <0.01 |  |  | |
|  | BMI | 0.98 (0.95-1.00) | | 0.04 | 1439 | | -- | -- |  |  | |
|  | Duration of diabetes | 1.11 (1.08-1.14) | | <0.01 | 1439 | | 1.07 (1.04-1.11) | <0.01 |  |  | |
|  | Age | 0.99 (0.97-1.02) | | 0.66 | 1439 | | 1.02 (0.99-1.05) | 0.13 |  |  | |
|  | IRSAD score | 1.00 (1.00-1.00) | | 0.52 | 1407 | | -- | -- |  |  | |
|  | Preexisting hypertension |  | |  | 1439 | |  |  |  |  | |
|  | No | REF | | REF |  | | REF | REF |  |  | |
|  | Yes | 2.06 (1.33-3.21) | | <0.01 |  | | 2.28 (1.42-3.65) | <0.01 |  |  | |
|  | Smoking during pregnancy |  | |  | 1439 | |  |  |  |  | |
|  | No | REF | | REF |  | | REF | REF |  |  | |
|  | Yes | 1.38 (0.86-2.23) | | 0.19 |  | | 1.53 (0.92-2.55) | 0.1 |  |  | |
|  | Region of maternal birth |  | |  | 1434 | |  |  |  |  | |
|  | Australia or New Zealand | REF | | REF |  | | -- | -- |  |  | |
|  | Asia | 0.57 (0.38-0.86) | | <0.01 |  | | -- | -- |  |  | |
|  | Europe | 0.75 (0.36-1.54) | | 0.43 |  | | -- | -- |  |  | |
|  | Americas | 1.18 (0.39-3.55) | | 0.76 |  | | -- | -- |  |  | |
|  | Africa | 0.48 (0.06-3.78) | | 0.49 |  | | -- | -- |  |  | |
|  | Oceania | 0.24 (0.03-1.81) | | 0.17 |  | | -- | -- |  |  | |
| **Preterm birth** | Diabetes type |  | |  | 1461 | |  |  | 1424 | 0.71 | |
|  | Type 2 | REF | | REF |  | | REF | REF |  |  | |
|  | Type 1 | 3.96 (3.07-5.11) | | <0.01 |  | | 3.67 (2.72-4.96) | <0.01 |  |  | |
|  | BMI | 0.98 (0.96-0.99) | | <0.01 | 1461 | | -- | -- |  |  | |
|  | Duration of diabetes | 1.11 (1.09-1.14) | | <0.01 | 1461 | | 1.06 (1.03-1.09) | <0.01 |  |  | |
|  | Age | 0.99 (0.97-1.01) | | 0.5 | 1461 | | 1.03 (1.01-1.06) | 0.01 |  |  | |
|  | IRSAD score | 1.00 (1.00-1.00) | | 0.19 | 1429 | | 1.00 (1.00-1.00) | <0.01 |  |  | |
|  | Preexisting hypertension |  | |  | 1461 | |  |  |  |  | |
|  | No | REF | | REF |  | | REF | REF |  |  | |
|  | Yes | 1.80 (1.22-2.64) | | <0.01 |  | | 2.09 (1.37-3.19) | <0.01 |  |  | |
|  | Smoking during pregnancy |  | |  | 1461 | |  |  |  |  | |
|  | No | REF | | REF |  | | -- | -- |  |  | |
|  | Yes | 1.23 (0.82-1.85) | | 0.32 |  | | -- | -- |  |  | |
|  | Region of maternal birth |  | |  | 1456 | |  |  |  |  | |
|  | Australia or New Zealand | REF | | REF |  | | -- | -- |  |  | |
|  | Asia | 0.45 (0.32-0.64) | | <0.01 |  | | -- | -- |  |  | |
|  | Europe | 0.81 (0.46-1.42) | | 0.46 |  | | -- | -- |  |  | |
|  | Americas | 0.26 (0.06-1.11) | | 0.07 |  | | -- | -- |  |  | |
|  | Africa | 1.36 (0.41-4.55) | | 0.62 |  | | -- | -- |  |  | |
|  | Oceania | 0.76 (0.28-2.06) | | 0.58 |  | | -- | -- |  |  | |
| **SGA** | Diabetes type |  | |  | 1461 | |  |  | 1424 | 0.63 | |
|  | Type 2 | REF | | REF |  | | REF | REF |  |  | |
|  | Type 1 | 0.35 (0.22-0.58) | | <0.01 |  | | 0.38 (0.22-0.65) | <0.01 |  |  | |
|  | BMI | 0.99 (0.96-1.02) | | 0.61 | 1461 | | 0.97 (0.94-1.00) | 0.08 |  |  | |
|  | Duration of diabetes | 0.93 (0.88-0.97) | | <0.01 | 1461 | | 0.96 (0.91-1.02) | 0.16 |  |  | |
|  | Age | 1.02 (0.98-1.06) | | 0.27 | 1461 | | -- | -- |  |  | |
|  | IRSAD score | 1.00 (1.00-1.00) | | 0.29 | 1429 | | -- | -- |  |  | |
|  | Preexisting hypertension |  | |  | 1461 | |  |  |  |  | |
|  | No | REF | | REF |  | | -- | -- |  |  | |
|  | Yes | 1.02 (0.52-2.02) | | 0.94 |  | | -- | -- |  |  | |
|  | Smoking during pregnancy |  | |  | 1461 | |  |  |  |  | |
|  | No | REF | | REF |  | | -- | -- |  |  | |
|  | Yes | 1.27 (0.67-2.37) | | 0.46 |  | | -- | -- |  |  | |
|  | Region of maternal birth |  | |  | 1456 | |  |  |  |  | |
|  | Australia or New Zealand | REF | | REF |  | | -- | -- |  |  | |
|  | Asia | 2.14 (1.40-3.26) | | <0.01 |  | | -- | -- |  |  | |
|  | Europe | 1.65 (0.72-3.75) | | 0.23 |  | | -- | -- |  |  | |
|  | Americas | 1.50 (0.34-6.56) | | 0.59 |  | | -- | -- |  |  | |
|  | Africa | 1.43 (0.18-11.31) | | 0.73 |  | | -- | -- |  |  | |
|  | Oceania | 1.50 (0.34-6.56) | | 0.59 |  | | -- | -- |  |  | |
| **LGA** | Diabetes type |  | |  | 1461 | |  |  | 1424 | 0.73 | |
|  | Type 2 | REF | | REF |  | | REF | REF |  |  | |
|  | Type 1 | 5.24 (4.13-6.63) | | <0.01 |  | | 5.14 (3.88-6.80) | <0.01 |  |  | |
|  | BMI | 0.98 (0.97-1.00) | | 0.054 | 1461 | | 1.02 (1.00-1.04) | 0.01 |  |  | |
|  | Duration of diabetes | 1.09 (1.06-1.11) | | <0.01 | 1461 | | -- | -- |  |  | |
|  | Age | 0.98 (0.96-1.00) | | 0.02 | 1461 | | -- | -- |  |  | |
|  | IRSAD score | 1.00 (1.00-1.00) | | 0.25 | 1429 | | -- | -- |  |  | |
|  | Preexisting hypertension |  | |  | 1461 | |  |  |  |  | |
|  | No | REF | | REF |  | | -- | -- |  |  | |
|  | Yes | 0.80 (0.53-1.19) | | 0.26 |  | | -- | -- |  |  | |
|  | Smoking during pregnancy |  | |  | 1461 | |  |  |  |  | |
|  | No | REF | | REF |  | | REF | REF |  |  | |
|  | Yes | 0.69 (0.46-1.04) | | 0.08 |  | | 0.71 (0.45-1.10) | 0.13 |  |  | |
|  | Region of maternal birth |  | |  | 1456 | |  |  |  |  | |
|  | Australia or New Zealand | REF | | REF |  | | REF | REF |  |  | |
|  | Asia | 0.30 (0.22-0.42) | | <0.01 |  | | 0.54 (0.37-0.77) | <0.01 |  |  | |
|  | Europe | 1.04 (0.64-1.69) | | 0.87 |  | | 1.35 (0.79-2.29) | 0.27 |  |  | |
|  | Americas | 0.71 (0.29-1.74) | | 0.45 |  | | 0.73 (0.27-2.01) | 0.54 |  |  | |
|  | Africa | 0.32 (0.07-1.49) | | 0.15 |  | | 0.37 (0.07-1.79) | 0.21 |  |  | |
|  | Oceania | 0.24 (0.07-0.82) | | 0.02 |  | | 0.49 (0.14-1.69) | 0.26 |  |  | |
| **Neonatal resuscitation** | Diabetes type |  | |  | 1461 | |  |  | 1424 | 0.58 | |
|  | Type 2 | REF | | REF |  | | REF | REF |  |  | |
|  | Type 1 | 1.53 (1.22-1.91) | | <0.01 |  | | 1.41 (1.07-1.86) | 0.02 |  |  | |
|  | BMI | 1.01 (1.00-1.03) | | 0.15 | 1461 | | 1.02 (1.00-1.04) | 0.02 |  |  | |
|  | Duration of diabetes | 1.04 (1.02-1.07) | | <0.01 | 1461 | | 1.03 (1.00-1.06) | 0.03 |  |  | |
|  | Age | 0.97 (0.96-0.99) | | 0.01 | 1461 | | 0.98 (0.96-1.01) | 0.15 |  |  | |
|  | IRSAD score | 1.00 (1.00-1.00) | | 0.052 | 1429 | | 1.00 (1.00-1.00) | 0.08 |  |  | |
|  | Preexisting hypertension |  | |  | 1461 | |  |  |  |  | |
|  | No | REF | | REF |  | | REF | REF |  |  | |
|  | Yes | 1.37 (0.94-1.99) | | 0.1 |  | | 1.40 (0.95-2.07) | 0.09 |  |  | |
|  | Smoking during pregnancy |  | |  | 1461 | |  |  |  |  | |
|  | No | REF | | REF |  | | -- | -- |  |  | |
|  | Yes | 1.35 (0.93-1.96) | | 0.12 |  | | -- | -- |  |  | |
|  | Region of maternal birth |  | |  | 1456 | |  |  |  |  | |
|  | Australia or New Zealand | REF | | REF |  | | -- | -- |  |  | |
|  | Asia | 0.68 (0.52-0.90) | | <0.01 |  | | -- | -- |  |  | |
|  | Europe | 0.84 (0.50-1.40) | | 0.5 |  | | -- | -- |  |  | |
|  | Americas | 1.06 (0.44-2.52) | | 0.9 |  | | -- | -- |  |  | |
|  | Africa | 0.99 (0.30-3.31) | | 0.99 |  | | -- | -- |  |  | |
|  | Oceania | 1.06 (0.44-2.52) | | 0.9 |  | | -- | -- |  |  | |
| **Neonatal hypoglycaemia** | Diabetes type |  | |  | 1440 | |  |  | 1403 | 0.68 | |
|  | Type 2 | REF | | REF |  | | REF | REF |  |  | |
|  | Type 1 | 3.76 (3.01-4.71) | | <0.01 |  | | 4.21 (3.32-5.34) | <0.01 |  |  | |
|  | BMI | 0.98 (0.96-0.99) | | <0.01 | 1440 | | -- | -- |  |  | |
|  | Duration of diabetes | 1.07 (1.05-1.09) | | <0.01 | 1440 | | -- | -- |  |  | |
|  | Age | 0.99 (0.97-1.01) | | 0.39 | 1440 | | 1.02 (1.00-1.04) | 0.04 |  |  | |
|  | IRSAD score | 1.00 (1.00-1.00) | | 0.49 | 1408 | | -- | -- |  |  | |
|  | Preexisting hypertension |  | |  | 1440 | |  |  |  |  | |
|  | No | REF | | REF |  | | REF | REF |  |  | |
|  | Yes | 1.53 (1.06-2.21) | | 0.02 |  | | 1.89 (1.27-2.80) | <0.01 |  |  | |
|  | Smoking during pregnancy |  | |  | 1440 | |  |  |  |  | |
|  | No | REF | | REF |  | | REF | REF |  |  | |
|  | Yes | 1.17 (0.81-1.68) | | 0.41 |  | | 1.42 (0.96-2.11) | 0.08 |  |  | |
|  | Region of maternal birth |  | |  | 1435 | |  |  |  |  | |
|  | Australia or New Zealand | REF | | REF |  | | -- | -- |  |  | |
|  | Asia | 0.68 (0.53-0.88) | | <0.01 |  | | -- | -- |  |  | |
|  | Europe | 1.24 (0.77-2.00) | | 0.37 |  | | -- | -- |  |  | |
|  | Americas | 0.84 (0.35-1.98) | | 0.69 |  | | -- | -- |  |  | |
|  | Africa | 0.40 (0.11-1.50) | | 0.18 |  | | -- | -- |  |  | |
|  | Oceania | 0.93 (0.40-2.15) | | 0.87 |  | | -- | -- |  |  | |
| **NICU admission** | Diabetes type |  | |  | 577 | |  |  | 560 | 0.69 | |
|  | Type 2 | REF | | REF |  | | REF | REF |  |  | |
|  | Type 1 | 3.15 (2.22-4.46) | | <0.01 |  | | 3.42 (2.23-5.24) | <0.01 |  |  | |
|  | BMI | 0.98 (0.96-1.00) | | 0.1 | 577 | | -- | -- |  |  | |
|  | Duration of diabetes | 1.09 (1.05-1.13) | | <0.01 | 577 | | 1.05 (1.00-1.09) | 0.03 |  |  | |
|  | Age | 1.01 (0.99-1.04) | | 0.34 | 577 | | 1.05 (1.02-1.08) | <0.01 |  |  | |
|  | IRSAD score | 1.00 (0.99-1.00) | | 0.02 | 564 | | 1.00 (0.99-1.00) | <0.01 |  |  | |
|  | Preexisting hypertension |  | |  | 577 | |  |  |  |  | |
|  | No | REF | | REF |  | | REF | REF |  |  | |
|  | Yes | 1.64 (0.96-2.79) | | 0.07 |  | | 1.87 (1.05-3.34) | 0.03 |  |  | |
|  | Smoking during pregnancy |  | |  | 577 | |  |  |  |  | |
|  | No | REF | | REF |  | | -- | -- |  |  | |
|  | Yes | 1.00 (0.61-1.65) | | 1 |  | | -- | -- |  |  | |
|  | Region of maternal birth |  | |  | 573 | |  |  |  |  | |
|  | Australia or New Zealand | REF | | REF |  | | -- | -- |  |  | |
|  | Asia | 0.68 (0.44-1.04) | | 0.07 |  | | -- | -- |  |  | |
|  | Europe | 1.36 (0.60-3.06) | | 0.46 |  | | -- | -- |  |  | |
|  | Americas | 1.60 (0.44-5.76) | | 0.47 |  | | -- | -- |  |  | |
|  | Africa | 1.07 (0.15-7.66) | | 0.95 |  | | -- | -- |  |  | |
|  | Oceania | 3.19 (0.64-16.07) | | 0.16 |  | | -- | -- |  |  | |
| **Neonatal hospital LOS > 10 days** | Diabetes type |  | |  | 1440 | |  |  | 1403 | 0.68 | |
|  | Type 2 | REF | | REF |  | | REF | REF |  |  | |
|  | Type 1 | 2.96 (2.17-4.02) | | <0.01 |  | | 2.45 (1.71-3.53) | <0.01 |  |  | |
|  | BMI | 0.98 (0.95-1.00) | | 0.03 | 1440 | | -- | -- |  |  | |
|  | Duration of diabetes | 1.11 (1.08-1.14) | | <0.01 | 1440 | | 1.07 (1.04-1.10) | <0.01 |  |  | |
|  | Age | 0.99 (0.97-1.02) | | 0.67 | 1440 | | 1.02 (0.99-1.05) | 0.13 |  |  | |
|  | IRSAD score | 1.00 (1.00-1.00) | | 0.52 | 1408 | | -- | -- |  |  | |
|  | Preexisting hypertension |  | |  | 1440 | |  |  |  |  | |
|  | No | REF | | REF |  | | REF | REF |  |  | |
|  | Yes | 2.07 (1.33-3.21) | | <0.01 |  | | 2.28 (1.43-3.65) | <0.01 |  |  | |
|  | Smoking during pregnancy |  | |  | 1440 | |  |  |  |  | |
|  | No | REF | | REF |  | | REF | REF |  |  | |
|  | Yes | 1.38 (0.86-2.23) | | 0.19 |  | | 1.54 (0.92-2.56) | 0.1 |  |  | |
|  | Region of maternal birth |  | |  | 1435 | |  |  |  |  | |
|  | Australia or New Zealand | REF | | REF |  | | -- | -- |  |  | |
|  | Asia | 0.57 (0.38-0.86) | | <0.01 |  | | -- | -- |  |  | |
|  | Europe | 0.75 (0.37-1.54) | | 0.43 |  | | -- | -- |  |  | |
|  | Americas | 1.18 (0.40-3.55) | | 0.76 |  | | -- | -- |  |  | |
|  | Africa | 0.48 (0.06-3.79) | | 0.49 |  | | -- | -- |  |  | |
|  | Oceania | 0.24 (0.03-1.81) | | 0.17 |  | | -- | -- |  |  | |
| **Stillbirth** | Diabetes type |  | |  | 1478 | |  |  | 1441 | 0.66 | |
|  | Type 2 | REF | | REF |  | | REF | REF |  |  | |
|  | Type 1 | 2.31 (0.87-6.11) | | 0.09 |  | | 2.85 (1.06-7.71) | 0.04 |  |  | |
|  | BMI | 0.99 (0.92-1.06) | | 0.74 | 1478 | | -- | -- |  |  | |
|  | Duration of diabetes | 1.04 (0.95-1.14) | | 0.36 | 1478 | | -- | -- |  |  | |
|  | Age | 0.95 (0.88-1.04) | | 0.3 | 1478 | | -- | -- |  |  | |
|  | IRSAD score | 0.99 (0.99-1.00) | | 0.1 | 1446 | | 0.99 (0.99-1.00) | 0.06 |  |  | |
|  | Preexisting hypertension |  | |  | 1478 | |  |  |  |  | |
|  | No | REF | | REF |  | | REF | REF |  |  | |
|  | Yes | 3.18 (1.02-9.90) | | 0.046 |  | | 3.56 (1.13-11.26) | 0.03 |  |  | |
|  | Smoking during pregnancy |  | |  | 1478 | |  |  |  |  | |
|  | No | REF | | REF |  | | -- | -- |  |  | |
|  | Yes | 0.64 (0.08-4.87) | | 0.67 |  | | -- | -- |  |  | |
|  | Region of maternal birth |  | |  | 1473 | |  |  |  |  | |
|  | Australia or New Zealand | REF | | REF |  | | -- | -- |  |  | |
|  | Asia | 0.20 (0.03-1.50) | | 0.12 |  | | -- | -- |  |  | |
|  | Europe | 0.00 (0.00-Inf) | | 0.99 |  | | -- | -- |  |  | |
|  | Americas | 0.00 (0.00-Inf) | | 0.99 |  | | -- | -- |  |  | |
|  | Africa | 0.00 (0.00-Inf) | | 1 |  | | -- | -- |  |  | |
|  | Oceania | 2.87 (0.36-22.67) | | 0.32 |  | | -- | -- |  |  | |
| Results are presented as odds ratios (OR) with 95% confidence intervals (CI), and p-values. The multivariable models were calculated using stepwise selection based on Akaike Information Criterion (AIC) with both forward and backward selection to determine the optimal set of predictors. -- indicates that the variable was dropped during stepwise selection using AIC. The multivariable models were internally validated using 10-fold cross-validated area under the receiver operating characteristic curve (AUC).  BMI, body mass index; IRSAD, index of relative socio-economic advantage and disadvantage; SGA, small for gestational age; LGA, large for gestational age; LOS, length of stay; NICU, neonatal intensive care. | | | | | | | | | | |  |

**Supplementary table 4: Maternal characteristics of all patients, by diabetes type and obesity status**

|  |  |  | **Type 1** | | **Type 2** | |  |
| --- | --- | --- | --- | --- | --- | --- | --- |
|  | **N** | **Overall**  N = 1478 | **Non-obesity** N = 448 | **Obesity** N = 120 | **Non-obesity** N = 440 | **Obesity** N = 470 | **p-value** |
| **Age (years), Median (IQR)** | 1478 | 31.0 (27.0, 34.0) | 30.0 (25.0, 33.0) | 28.0 (23.0, 31.5) | 31.0 (28.0, 35.0) | 31.0 (28.0, 36.0) | <0.001^1^ |
| **Glycaemic control (HbA1c, %)** |  |  |  |  |  |  |  |
| Pre-Pregnancy, Median (IQR) | 35 | 8.7 (7.7, 9.8) | 8.7 (7.8, 11.4) | 8.7 (7.5, 9.7) | 6.7 (5.7, 7.7) | 10.4 (8.6, 12.8) | 0.189^1^ |
| Missing (%) |  | 1443 (98%) | 425 (95%) | 113 (94%) | 438 (100%) | 467 (99%) |  |
| <20 weeks gestation, Median (IQR) | 238 | 6.4 (5.6, 7.5) | 6.8 (6.1, 7.8) | 7.1 (6.3, 7.9) | 6.5 (5.2, 7.5) | 6.0 (5.2, 6.9) | <0.001^1^ |
| Missing (%) |  | 1240 (84%) | 366 (82%) | 98 (82%) | 388 (88%) | 388 (83%) |  |
| Trimester 1, Median (IQR) | 169 | 6.7 (5.9, 7.7) | 6.9 (6.1, 8.0) | 7.1 (6.8, 8.0) | 6.6 (5.5, 7.7) | 6.1 (5.3, 7.2) | 0.005^1^ |
| Missing (%) |  | 1309 (89%) | 390 (87%) | 100 (83%) | 400 (91%) | 419 (89%) |  |
| Trimester 2, Median (IQR) | 120 | 5.9 (5.3, 6.7) | 6.1 (5.5, 6.8) | 6.3 (6.1, 6.8) | 5.3 (4.9, 6.7) | 5.6 (5.1, 6.0) | 0.007^1^ |
| Missing (%) |  | 1358 (92%) | 399 (89%) | 112 (93%) | 417 (95%) | 430 (91%) |  |
| Trimester 3, Median (IQR) | 88 | 6.3 (5.5, 6.8) | 6.4 (5.7, 7.0) | 6.4 (6.0, 6.7) | 6.4 (5.5, 6.6) | 5.9 (5.3, 6.2) | 0.192^1^ |
| Missing (%) |  | 1390 (94%) | 408 (91%) | 106 (88%) | 427 (97%) | 449 (96%) |  |
| Post-Pregnancy, Mean (SD) | 36 | 8.4 (2.4) | 8.4 (2.1) | 7.7 (1.2) | 11.3 (2.4) | 7.2 (2.7) | 0.127^2^ |
| Missing (%) |  | 1442 (98%) | 429 (96%) | 114 (95%) | 436 (99%) | 463 (99%) |  |
| **Duration of diabetes (years), Median (IQR)** | 1478 | 1.7 (0.0, 6.1) | 5.8 (0.3, 12.2) | 6.9 (1.1, 12.6) | 1.0 (0.0, 3.0) | 1.2 (0.0, 3.2) | <0.001^1^ |
| **Maternal weight (kg), Median (IQR)** | 1478 | 75.0 (65.0, 91.0) | 65.0 (60.0, 72.0) | 92.0 (84.0, 105.5) | 69.0 (61.0, 74.0) | 95.0 (86.0, 110.0) | <0.001^1^ |
| **Maternal height (cm), Median (IQR)** | 1478 | 164.0 (160.0, 169.0) | 165.0 (160.0, 170.0) | 165.0 (162.0, 170.0) | 163.0 (158.0, 167.0) | 164.0 (160.0, 169.0) | <0.001^1^ |
| **BMI (kg/m²), Median (IQR)** | 1478 | 28.2 (23.9, 33.3) | 23.9 (22.0, 26.1) | 34.3 (31.2, 37.6) | 25.7 (23.4, 28.0) | 35.1 (32.5, 39.5) | <0.001^1^ |
| **BMI Category (kg/m²), n (%)** | 1478 |  |  |  |  |  | <0.001^3^ |
| Normal weight (BMI 18.5-24.9) |  | 479 (32.4%) | 290 (64.7%) | 0 (0.0%) | 189 (43.0%) | 0 (0.0%) |  |
| Overweight (BMI 25-29.9) |  | 409 (27.7%) | 158 (35.3%) | 0 (0.0%) | 251 (57.0%) | 0 (0.0%) |  |
| Obesity (BMI 30+) |  | 590 (39.9%) | 0 (0.0%) | 120 (100.0%) | 0 (0.0%) | 470 (100.0%) |  |
| **Antenatal care received, n (%)** | 1478 | 1468 (99.3%) | 445 (99.3%) | 119 (99.2%) | 440 (100.0%) | 464 (98.7%) | 0.086^4^ |
| **Smoking during pregnancy, n (%)** | 1478 | 131 (8.9%) | 31 (6.9%) | 7 (5.8%) | 38 (8.6%) | 55 (11.7%) | 0.043^3^ |
| **Pre-existing hypertension, n (%)** | 1478 | 133 (9.0%) | 20 (4.5%) | 16 (13.3%) | 24 (5.5%) | 73 (15.5%) | <0.001^3^ |
| **IRSAD Score, Median (IQR)** | 1446 | 985.0 (938.0, 1039.0) | 1006.0 (949.0, 1064.0) | 982.0 (941.5, 1033.5) | 987.0 (933.0, 1039.0) | 969.0 (928.0, 1022.0) | <0.001^1^ |
| Missing (%) |  | 32 (2.2%) | 11 (2.5%) | 0 (0%) | 8 (1.8%) | 13 (2.8%) |  |
| **IRSAD Quintile, n (%)** | 1446 |  |  |  |  |  | <0.001^3^ |
| Most disadvantaged quintile |  | 343 (23.7%) | 79 (18.1%) | 27 (22.5%) | 111 (25.7%) | 126 (27.6%) |  |
| 2 |  | 367 (25.4%) | 98 (22.4%) | 34 (28.3%) | 97 (22.5%) | 138 (30.2%) |  |
| 3 |  | 287 (19.8%) | 94 (21.5%) | 27 (22.5%) | 84 (19.4%) | 82 (17.9%) |  |
| 4 |  | 268 (18.5%) | 83 (19.0%) | 23 (19.2%) | 90 (20.8%) | 72 (15.8%) |  |
| Most advantaged quintile |  | 181 (12.5%) | 83 (19.0%) | 9 (7.5%) | 50 (11.6%) | 39 (8.5%) |  |
| Missing (%) |  | 32 (2.2%) | 11 (2.5%) | 0 (0%) | 8 (1.8%) | 13 (2.8%) |  |
| **Region of maternal birth, n (%)** | 1473 |  |  |  |  |  | <0.001^3^ |
| Australia and New Zealand |  | 1004 (68.2%) | 373 (83.4%) | 111 (92.5%) | 194 (44.4%) | 326 (69.5%) |  |
| Asia |  | 336 (22.8%) | 35 (7.8%) | 5 (4.2%) | 193 (44.2%) | 103 (22.0%) |  |
| Europe |  | 74 (5.0%) | 25 (5.6%) | 4 (3.3%) | 26 (5.9%) | 19 (4.1%) |  |
| Oceania |  | 24 (1.6%) | 1 (0.2%) | 0 (0.0%) | 11 (2.5%) | 12 (2.6%) |  |
| Americas |  | 23 (1.6%) | 9 (2.0%) | 0 (0.0%) | 10 (2.3%) | 4 (0.9%) |  |
| Africa |  | 12 (0.8%) | 4 (0.9%) | 0 (0.0%) | 3 (0.7%) | 5 (1.1%) |  |
| Missing (%) |  | 5 (0.3%) | 1 (0.2%) | 0 (0%) | 3 (0.7%) | 1 (0.2%) |  |
| ^1^Kruskal-Wallis rank sum test | | | | | | | |
| ^2^One-way analysis of means (not assuming equal variances) | | | | | | | |
| ^3^Pearson's Chi-squared test | | | | | | | |
| ^4^Fisher's exact test | | | | | | | |
| Continuous: non-normal = median (IQR), Kruskal-Wallis; normal = mean (SD), ANOVA Categorical: n (%), Chi-square/Fisher | | | | | | | |

**Supplementary Table 5: Univariable and multivariable logistic regression models of perinatal outcomes, stratified by type 1 and type 2 diabetes**

|  | | **Type 1** | | | | | | | **Type 2** | | | | | | |
| --- | --- | --- | --- | --- | --- | --- | --- | --- | --- | --- | --- | --- | --- | --- | --- |
|  | | **Univariable** | | | **Multivariable** | | | | **Univariable** | | | **Multivariable** | | | |
| **Outcome** | **Predictor** | **OR (95% CI)** | **p-value** | **N** | **OR (95% CI)** | **p-value** | **N** | **AUC** | **OR (95% CI)** | **p-value** | **N** | **OR (95% CI)** | **p-value** | **N** | **AUC** |
| **Preeclampsia** | BMI category |  |  | 568 |  |  | 556 | 0.68 |  |  | 910 |  |  | 885 | 0.68 |
|  | Normal weight | REF | REF |  | -- | -- |  |  | REF | REF |  | -- | -- |  |  |
|  | Overweight | 1.57 (0.81-3.02) | 0.18 |  | -- | -- |  |  | 1.27 (0.45-3.55) | 0.65 |  | -- | -- |  |  |
|  | Obesity | 1.61 (0.79-3.27) | 0.19 |  | -- | -- |  |  | 2.61 (1.08-6.29) | 0.03 |  | -- | -- |  |  |
|  | Duration of diabetes | 1.07 (1.02-1.13) | 0.01 | 568 | 1.07 (1.01-1.13) | 0.01 |  |  | 1.03 (0.95-1.12) | 0.44 | 910 | -- | -- |  |  |
|  | Age | 0.99 (0.94-1.05) | 0.79 | 568 | -- | -- |  |  | 1.03 (0.98-1.08) | 0.25 | 910 | -- | -- |  |  |
|  | IRSAD score | 1.00 (0.99-1.00) | 0.14 | 557 | -- | -- |  |  | 1.00 (1.00-1.00) | 0.91 | 889 | -- | -- |  |  |
|  | Preexisting hypertension |  |  | 568 |  |  |  |  |  |  | 910 |  |  |  |  |
|  | No | REF | REF |  | REF | REF |  |  | REF | REF |  | REF | REF |  |  |
|  | Yes | 5.00 (2.30-10.88) | <0.01 |  | 4.84 (2.20-10.63) | <0.01 |  |  | 8.89 (4.91-16.07) | <0.01 |  | 9.01 (4.97-16.32) | <0.01 |  |  |
|  | Smoking during pregnancy |  |  | 568 |  |  |  |  |  |  | 910 |  |  |  |  |
|  | No | REF | REF |  | -- | -- |  |  | REF | REF |  | -- | -- |  |  |
|  | Yes | 1.13 (0.38-3.32) | 0.82 |  | -- | -- |  |  | 1.36 (0.60-3.12) | 0.46 |  | -- | -- |  |  |
|  | Region of maternal birth |  |  | 567 |  |  |  |  |  |  | 906 |  |  |  |  |
|  | Australia or New Zealand | REF | REF |  | -- | -- |  |  | REF | REF |  | -- | -- |  |  |
|  | Asia | 0.46 (0.11-1.96) | 0.29 |  | -- | -- |  |  | 0.48 (0.24-0.99) | 0.048 |  | -- | -- |  |  |
|  | Europe | 0.64 (0.15-2.79) | 0.55 |  | -- | -- |  |  | 2.13 (0.84-5.39) | 0.11 |  | -- | -- |  |  |
|  | Americas | 0.00 (0.00-Inf) | 0.99 |  | -- | -- |  |  | 0.00 (0.00-Inf) | 0.99 |  | -- | -- |  |  |
|  | Africa | 0.00 (0.00-Inf) | 0.99 |  | -- | -- |  |  | 0.00 (0.00-Inf) | 0.99 |  | -- | -- |  |  |
|  | Oceania | 0.00 (0.00-Inf) | 1 |  | -- | -- |  |  | 1.32 (0.30-5.87) | 0.72 |  | -- | -- |  |  |
| **Gestational hypertension** | BMI category |  |  | 568 |  |  | 556 | 0.54 |  |  | 910 |  |  | 885 | 0.64 |
|  | Normal weight | REF | REF |  | -- | -- |  |  | REF | REF |  | REF | REF |  |  |
|  | Overweight | 1.00 (0.48-2.08) | 1 |  | -- | -- |  |  | 6.37 (1.44-28.09) | 0.01 |  | 5.93 (1.34-26.29) | 0.02 |  |  |
|  | Obesity | 1.11 (0.51-2.42) | 0.8 |  | -- | -- |  |  | 7.52 (1.79-31.66) | <0.01 |  | 5.88 (1.38-25.15) | 0.02 |  |  |
|  | Duration of diabetes | 0.99 (0.93-1.05) | 0.68 | 568 | -- | -- |  |  | 0.98 (0.89-1.07) | 0.65 | 910 | -- | -- |  |  |
|  | Age | 1.00 (0.95-1.06) | 0.95 | 568 | -- | -- |  |  | 1.03 (0.98-1.08) | 0.24 | 910 | 1.05 (1.00-1.11) | 0.06 |  |  |
|  | IRSAD score | 1.00 (1.00-1.00) | 0.99 | 557 | -- | -- |  |  | 1.00 (0.99-1.00) | 0.37 | 889 | -- | -- |  |  |
|  | Preexisting hypertension |  |  | 568 |  |  |  |  |  |  | 910 |  |  |  |  |
|  | No | REF | REF |  | -- | -- |  |  | REF | REF |  | -- | -- |  |  |
|  | Yes | 1.54 (0.52-4.58) | 0.44 |  | -- | -- |  |  | 1.30 (0.57-2.96) | 0.54 |  | -- | -- |  |  |
|  | Smoking during pregnancy |  |  | 568 |  |  |  |  |  |  | 910 |  |  |  |  |
|  | No | REF | REF |  | REF | REF |  |  | REF | REF |  | -- | -- |  |  |
|  | Yes | 0.00 (0.00-Inf) | 0.99 |  | 0.00 (0.00-Inf) | 0.99 |  |  | 1.13 (0.47-2.72) | 0.79 |  | -- | -- |  |  |
|  | Region of maternal birth |  |  | 567 |  |  |  |  |  |  | 906 |  |  |  |  |
|  | Australia or New Zealand | REF | REF |  | -- | -- |  |  | REF | REF |  | REF | REF |  |  |
|  | Asia | 0.60 (0.14-2.59) | 0.49 |  | -- | -- |  |  | 0.46 (0.23-0.92) | 0.03 |  | 0.51 (0.25-1.04) | 0.07 |  |  |
|  | Europe | 0.41 (0.05-3.09) | 0.38 |  | -- | -- |  |  | 0.27 (0.04-2.04) | 0.2 |  | 0.26 (0.03-1.95) | 0.19 |  |  |
|  | Americas | 1.43 (0.17-11.75) | 0.74 |  | -- | -- |  |  | 0.00 (0.00-Inf) | 0.99 |  | 0.00 (0.00-Inf) | 0.99 |  |  |
|  | Africa | 0.00 (0.00-Inf) | 0.99 |  | -- | -- |  |  | 1.71 (0.21-14.32) | 0.62 |  | 1.56 (0.18-13.30) | 0.68 |  |  |
|  | Oceania | 0.00 (0.00-Inf) | 0.99 |  | -- | -- |  |  | 0.00 (0.00-Inf) | 0.99 |  | 0.00 (0.00-Inf) | 0.99 |  |  |
| **Postpartum haemorrhage** | BMI category |  |  | 566 |  |  | 554 | 0.58 |  |  | 910 |  |  | 885 | 0.58 |
|  | Normal weight | REF | REF |  | -- | -- |  |  | REF | REF |  | REF | REF |  |  |
|  | Overweight | 0.98 (0.63-1.54) | 0.94 |  | -- | -- |  |  | 1.30 (0.75-2.25) | 0.34 |  | 1.30 (0.74-2.27) | 0.36 |  |  |
|  | Obesity | 0.79 (0.47-1.32) | 0.37 |  | -- | -- |  |  | 1.79 (1.10-2.90) | 0.02 |  | 1.95 (1.18-3.23) | <0.01 |  |  |
|  | Duration of diabetes | 1.04 (1.01-1.08) | 0.02 | 566 | 1.04 (1.00-1.08) | 0.03 |  |  | 1.05 (1.00-1.10) | 0.053 | 910 | 1.06 (1.00-1.11) | 0.04 |  |  |
|  | Age | 0.99 (0.96-1.03) | 0.74 | 566 | -- | -- |  |  | 1.00 (0.97-1.03) | 0.86 | 910 | -- | -- |  |  |
|  | IRSAD score | 1.00 (1.00-1.00) | 0.64 | 555 | -- | -- |  |  | 1.00 (1.00-1.00) | 0.35 | 889 | -- | -- |  |  |
|  | Preexisting hypertension |  |  | 566 |  |  |  |  |  |  | 910 |  |  |  |  |
|  | No | REF | REF |  | REF | REF |  |  | REF | REF |  | -- | -- |  |  |
|  | Yes | 0.38 (0.13-1.09) | 0.07 |  | 0.37 (0.13-1.06) | 0.06 |  |  | 1.15 (0.67-1.96) | 0.61 |  | -- | -- |  |  |
|  | Smoking during pregnancy |  |  | 566 |  |  |  |  |  |  | 910 |  |  |  |  |
|  | No | REF | REF |  | -- | -- |  |  | REF | REF |  | -- | -- |  |  |
|  | Yes | 1.14 (0.54-2.41) | 0.73 |  | -- | -- |  |  | 1.22 (0.71-2.09) | 0.47 |  | -- | -- |  |  |
|  | Region of maternal birth |  |  | 565 |  |  |  |  |  |  | 906 |  |  |  |  |
|  | Australia or New Zealand | REF | REF |  | -- | -- |  |  | REF | REF |  | REF | REF |  |  |
|  | Asia | 1.02 (0.48-2.15) | 0.97 |  | -- | -- |  |  | 0.89 (0.61-1.30) | 0.54 |  | 1.19 (0.78-1.81) | 0.43 |  |  |
|  | Europe | 0.80 (0.32-2.00) | 0.63 |  | -- | -- |  |  | 2.07 (1.06-4.05) | 0.03 |  | 2.40 (1.21-4.76) | 0.01 |  |  |
|  | Americas | 0.38 (0.05-3.09) | 0.37 |  | -- | -- |  |  | 1.25 (0.34-4.59) | 0.73 |  | 1.65 (0.44-6.17) | 0.46 |  |  |
|  | Africa | 0.00 (0.00-Inf) | 0.98 |  | -- | -- |  |  | 0.66 (0.08-5.41) | 0.69 |  | 0.70 (0.08-5.81) | 0.74 |  |  |
|  | Oceania | 0.00 (0.00-Inf) | 0.99 |  | -- | -- |  |  | 0.21 (0.03-1.57) | 0.13 |  | 0.22 (0.03-1.66) | 0.14 |  |  |
| **Caesarean section** | BMI category |  |  | 568 |  |  | 556 | 0.61 |  |  | 910 |  |  | 885 | 0.65 |
|  | Normal weight | REF | REF |  | -- | -- |  |  | REF | REF |  | REF | REF |  |  |
|  | Overweight | 1.08 (0.71-1.65) | 0.71 |  | -- | -- |  |  | 1.98 (1.35-2.90) | <0.01 |  | 1.95 (1.31-2.91) | <0.01 |  |  |
|  | Obesity | 1.19 (0.75-1.91) | 0.46 |  | -- | -- |  |  | 2.33 (1.65-3.30) | <0.01 |  | 2.29 (1.59-3.30) | <0.01 |  |  |
|  | Duration of diabetes | 1.04 (1.00-1.07) | 0.03 | 568 | 1.06 (1.02-1.09) | <0.01 |  |  | 0.99 (0.95-1.03) | 0.62 | 910 | -- | -- |  |  |
|  | Age | 1.06 (1.02-1.09) | <0.01 | 568 | 1.06 (1.02-1.10) | <0.01 |  |  | 1.08 (1.06-1.11) | <0.01 | 910 | 1.08 (1.05-1.11) | <0.01 |  |  |
|  | IRSAD score | 1.00 (1.00-1.00) | 0.07 | 557 | -- | -- |  |  | 1.00 (1.00-1.00) | 0.78 | 889 | -- | -- |  |  |
|  | Preexisting hypertension |  |  | 568 |  |  |  |  |  |  | 910 |  |  |  |  |
|  | No | REF | REF |  | -- | -- |  |  | REF | REF |  | REF | REF |  |  |
|  | Yes | 1.60 (0.71-3.59) | 0.25 |  | -- | -- |  |  | 2.26 (1.41-3.62) | <0.01 |  | 1.52 (0.92-2.48) | 0.1 |  |  |
|  | Smoking during pregnancy |  |  | 568 |  |  |  |  |  |  | 910 |  |  |  |  |
|  | No | REF | REF |  | -- | -- |  |  | REF | REF |  | -- | -- |  |  |
|  | Yes | 0.75 (0.38-1.49) | 0.41 |  | -- | -- |  |  | 0.88 (0.57-1.35) | 0.55 |  | -- | -- |  |  |
|  | Region of maternal birth |  |  | 567 |  |  |  |  |  |  | 906 |  |  |  |  |
|  | Australia or New Zealand | REF | REF |  | REF | REF |  |  | REF | REF |  | -- | -- |  |  |
|  | Asia | 0.68 (0.35-1.32) | 0.25 |  | 0.73 (0.36-1.45) | 0.37 |  |  | 1.24 (0.93-1.66) | 0.14 |  | -- | -- |  |  |
|  | Europe | 3.93 (1.17-13.22) | 0.03 |  | 5.38 (1.23-23.49) | 0.02 |  |  | 0.93 (0.50-1.71) | 0.81 |  | -- | -- |  |  |
|  | Americas | 0.91 (0.22-3.69) | 0.89 |  | 0.75 (0.17-3.27) | 0.7 |  |  | 0.45 (0.15-1.37) | 0.16 |  | -- | -- |  |  |
|  | Africa | 0.45 (0.06-3.26) | 0.43 |  | 0.48 (0.07-3.52) | 0.47 |  |  | 0.81 (0.20-3.29) | 0.77 |  | -- | -- |  |  |
|  | Oceania | 353346.29 (0.00-Inf) | 0.98 |  | 277146.76 (0.00-Inf) | 0.98 |  |  | 1.26 (0.54-2.97) | 0.59 |  | -- | -- |  |  |
| **Maternal hospital LOS > 10 days** | BMI category |  |  | 553 |  |  | 541 | 0.65 |  |  | 886 |  |  | 861 | 0.62 |
|  | Normal weight | REF | REF |  | REF | REF |  |  | REF | REF |  | REF | REF |  |  |
|  | Overweight | 0.68 (0.41-1.13) | 0.13 |  | 0.70 (0.41-1.19) | 0.18 |  |  | 2.55 (1.17-5.53) | 0.02 |  | 2.43 (1.11-5.33) | 0.03 |  |  |
|  | Obesity | 1.41 (0.86-2.29) | 0.17 |  | 1.31 (0.79-2.20) | 0.3 |  |  | 1.87 (0.89-3.94) | 0.1 |  | 1.61 (0.75-3.46) | 0.22 |  |  |
|  | Duration of diabetes | 1.08 (1.04-1.12) | <0.01 | 553 | 1.07 (1.03-1.12) | <0.01 |  |  | 1.06 (1.00-1.13) | 0.06 | 886 | 1.06 (0.99-1.13) | 0.09 |  |  |
|  | Age | 0.99 (0.95-1.03) | 0.56 | 553 | -- | -- |  |  | 1.05 (1.01-1.10) | 0.02 | 886 | 1.04 (1.00-1.09) | 0.06 |  |  |
|  | IRSAD score | 1.00 (0.99-1.00) | 0.03 | 542 | -- | -- |  |  | 1.00 (1.00-1.00) | 0.92 | 865 | -- | -- |  |  |
|  | Preexisting hypertension |  |  | 553 |  |  |  |  |  |  | 886 |  |  |  |  |
|  | No | REF | REF |  | REF | REF |  |  | REF | REF |  | REF | REF |  |  |
|  | Yes | 2.85 (1.41-5.76) | <0.01 |  | 2.50 (1.20-5.24) | 0.01 |  |  | 2.26 (1.23-4.16) | <0.01 |  | 1.87 (0.98-3.58) | 0.06 |  |  |
|  | Smoking during pregnancy |  |  | 553 |  |  |  |  |  |  | 886 |  |  |  |  |
|  | No | REF | REF |  | REF | REF |  |  | REF | REF |  | -- | -- |  |  |
|  | Yes | 2.46 (1.24-4.88) | <0.01 |  | 2.34 (1.16-4.73) | 0.02 |  |  | 1.00 (0.46-2.15) | 1 |  | -- | -- |  |  |
|  | Region of maternal birth |  |  | 552 |  |  |  |  |  |  | 882 |  |  |  |  |
|  | Australia or New Zealand | REF | REF |  | -- | -- |  |  | REF | REF |  | -- | -- |  |  |
|  | Asia | 0.35 (0.12-1.02) | 0.054 |  | -- | -- |  |  | 1.18 (0.71-1.95) | 0.52 |  | -- | -- |  |  |
|  | Europe | 0.51 (0.17-1.50) | 0.22 |  | -- | -- |  |  | 1.41 (0.53-3.78) | 0.49 |  | -- | -- |  |  |
|  | Americas | 1.59 (0.39-6.50) | 0.51 |  | -- | -- |  |  | 0.92 (0.12-7.26) | 0.94 |  | -- | -- |  |  |
|  | Africa | 0.00 (0.00-Inf) | 0.98 |  | -- | -- |  |  | 1.57 (0.19-13.14) | 0.67 |  | -- | -- |  |  |
|  | Oceania | 0.00 (0.00-Inf) | 0.99 |  | -- | -- |  |  | 0.52 (0.07-4.01) | 0.53 |  | -- | -- |  |  |
| **Preterm birth** | BMI category |  |  | 558 |  |  | 546 | 0.61 |  |  | 903 |  |  | 878 | 0.60 |
|  | Normal weight | REF | REF |  | -- | -- |  |  | REF | REF |  | REF | REF |  |  |
|  | Overweight | 0.90 (0.60-1.35) | 0.61 |  | -- | -- |  |  | 2.19 (1.17-4.09) | 0.01 |  | 2.10 (1.11-3.97) | 0.02 |  |  |
|  | Obesity | 1.26 (0.82-1.96) | 0.29 |  | -- | -- |  |  | 2.05 (1.14-3.68) | 0.02 |  | 1.81 (0.99-3.31) | 0.052 |  |  |
|  | Duration of diabetes | 1.06 (1.03-1.09) | <0.01 | 558 | 1.05 (1.02-1.09) | <0.01 |  |  | 1.07 (1.01-1.13) | 0.01 | 903 | 1.06 (1.01-1.12) | 0.02 |  |  |
|  | Age | 1.00 (0.97-1.03) | 0.94 | 558 | 1.03 (0.99-1.07) | 0.1 |  |  | 1.05 (1.01-1.09) | <0.01 | 903 | 1.04 (1.00-1.08) | 0.045 |  |  |
|  | IRSAD score | 1.00 (0.99-1.00) | <0.01 | 547 | 1.00 (0.99-1.00) | <0.01 |  |  | 1.00 (1.00-1.00) | 0.33 | 882 | -- | -- |  |  |
|  | Preexisting hypertension |  |  | 558 |  |  |  |  |  |  | 903 |  |  |  |  |
|  | No | REF | REF |  | REF | REF |  |  | REF | REF |  | REF | REF |  |  |
|  | Yes | 2.21 (1.10-4.42) | 0.03 |  | 1.91 (0.94-3.89) | 0.07 |  |  | 2.40 (1.44-4.00) | <0.01 |  | 1.92 (1.12-3.30) | 0.02 |  |  |
|  | Smoking during pregnancy |  |  | 558 |  |  |  |  |  |  | 903 |  |  |  |  |
|  | No | REF | REF |  | -- | -- |  |  | REF | REF |  | -- | -- |  |  |
|  | Yes | 1.45 (0.75-2.82) | 0.27 |  | -- | -- |  |  | 1.48 (0.84-2.60) | 0.18 |  | -- | -- |  |  |
|  | Region of maternal birth |  |  | 557 |  |  |  |  |  |  | 899 |  |  |  |  |
|  | Australia or New Zealand | REF | REF |  | REF | REF |  |  | REF | REF |  | -- | -- |  |  |
|  | Asia | 0.20 (0.08-0.52) | <0.01 |  | 0.26 (0.10-0.68) | <0.01 |  |  | 1.12 (0.74-1.69) | 0.58 |  | -- | -- |  |  |
|  | Europe | 0.73 (0.33-1.61) | 0.44 |  | 1.05 (0.45-2.44) | 0.91 |  |  | 1.21 (0.52-2.82) | 0.66 |  | -- | -- |  |  |
|  | Americas | 0.40 (0.08-1.94) | 0.25 |  | 0.52 (0.10-2.75) | 0.44 |  |  | 0.00 (0.00-Inf) | 0.98 |  | -- | -- |  |  |
|  | Africa | 0.46 (0.05-4.52) | 0.51 |  | 0.47 (0.05-4.71) | 0.52 |  |  | 3.94 (0.92-16.91) | 0.06 |  | -- | -- |  |  |
|  | Oceania | 1086204.78 (0.00-Inf) | 0.98 |  | 1079322.66 (0.00-Inf) | 0.98 |  |  | 1.46 (0.48-4.45) | 0.5 |  | -- | -- |  |  |
| **SGA** | BMI category |  |  | 558 |  |  | 546 | 0.62 |  |  | 903 |  |  | 878 | -- |
|  | Normal weight | REF | REF |  | -- | -- |  |  | REF | REF |  | -- | -- |  |  |
|  | Overweight | 1.17 (0.44-3.08) | 0.76 |  | -- | -- |  |  | 1.25 (0.68-2.30) | 0.46 |  | -- | -- |  |  |
|  | Obesity | 0.65 (0.18-2.37) | 0.51 |  | -- | -- |  |  | 0.84 (0.47-1.49) | 0.55 |  | -- | -- |  |  |
|  | Duration of diabetes | 0.96 (0.89-1.04) | 0.34 | 558 | -- | -- |  |  | 0.96 (0.89-1.04) | 0.3 | 903 | -- | -- |  |  |
|  | Age | 1.01 (0.93-1.10) | 0.86 | 558 | -- | -- |  |  | 1.00 (0.96-1.04) | 0.94 | 903 | -- | -- |  |  |
|  | IRSAD score | 1.00 (0.99-1.00) | 0.39 | 547 | -- | -- |  |  | 1.00 (1.00-1.00) | 0.84 | 882 | -- | -- |  |  |
|  | Preexisting hypertension |  |  | 558 |  |  |  |  |  |  | 903 |  |  |  |  |
|  | No | REF | REF |  | -- | -- |  |  | REF | REF |  | -- | -- |  |  |
|  | Yes | 0.74 (0.10-5.70) | 0.77 |  | -- | -- |  |  | 0.95 (0.46-1.97) | 0.89 |  | -- | -- |  |  |
|  | Smoking during pregnancy |  |  | 558 |  |  |  |  |  |  | 903 |  |  |  |  |
|  | No | REF | REF |  | -- | -- |  |  | REF | REF |  | -- | -- |  |  |
|  | Yes | 1.46 (0.33-6.56) | 0.62 |  | -- | -- |  |  | 1.11 (0.56-2.24) | 0.76 |  | -- | -- |  |  |
|  | Region of maternal birth |  |  | 557 |  |  |  |  |  |  | 899 |  |  |  |  |
|  | Australia or New Zealand | REF | REF |  | REF | REF |  |  | REF | REF |  | -- | -- |  |  |
|  | Asia | 4.37 (1.50-12.76) | <0.01 |  | 4.43 (1.52-12.96) | <0.01 |  |  | 1.44 (0.90-2.30) | 0.13 |  | -- | -- |  |  |
|  | Europe | 0.00 (0.00-Inf) | 0.99 |  | 0.00 (0.00-Inf) | 0.99 |  |  | 1.97 (0.83-4.68) | 0.12 |  | -- | -- |  |  |
|  | Americas | 0.00 (0.00-Inf) | 0.99 |  | 0.00 (0.00-Inf) | 0.99 |  |  | 1.78 (0.39-8.24) | 0.46 |  | -- | -- |  |  |
|  | Africa | 0.00 (0.00-Inf) | 1 |  | 0.00 (0.00-Inf) | 1 |  |  | 1.53 (0.18-12.75) | 0.69 |  | -- | -- |  |  |
|  | Oceania | 3538853060.41 (0.00-Inf) | 1 |  | 3484883620.91 (0.00-Inf) | 1 |  |  | 0.51 (0.07-3.89) | 0.52 |  | -- | -- |  |  |
| **LGA** | BMI category |  |  | 558 |  |  | 546 | 0.59 |  |  | 903 |  |  | 878 | 0.57 |
|  | Normal weight | REF | REF |  | -- | -- |  |  | REF | REF |  | REF | REF |  |  |
|  | Overweight | 0.89 (0.60-1.31) | 0.55 |  | -- | -- |  |  | 1.15 (0.67-1.96) | 0.62 |  | 1.14 (0.66-1.96) | 0.65 |  |  |
|  | Obesity | 1.08 (0.70-1.66) | 0.73 |  | -- | -- |  |  | 1.78 (1.11-2.84) | 0.02 |  | 1.82 (1.13-2.93) | 0.01 |  |  |
|  | Duration of diabetes | 1.02 (0.99-1.05) | 0.24 | 558 | -- | -- |  |  | 1.00 (0.95-1.06) | 0.94 | 903 | -- | -- |  |  |
|  | Age | 0.99 (0.96-1.03) | 0.68 | 558 | -- | -- |  |  | 1.03 (0.99-1.06) | 0.11 | 903 | 1.03 (1.00-1.06) | 0.1 |  |  |
|  | IRSAD score | 1.00 (1.00-1.00) | 0.89 | 547 | -- | -- |  |  | 1.00 (1.00-1.00) | 0.31 | 882 | -- | -- |  |  |
|  | Preexisting hypertension |  |  | 558 |  |  |  |  |  |  | 903 |  |  |  |  |
|  | No | REF | REF |  | REF | REF |  |  | REF | REF |  | -- | -- |  |  |
|  | Yes | 0.36 (0.17-0.75) | <0.01 |  | 0.33 (0.16-0.70) | <0.01 |  |  | 1.69 (1.03-2.76) | 0.04 |  | -- | -- |  |  |
|  | Smoking during pregnancy |  |  | 558 |  |  |  |  |  |  | 903 |  |  |  |  |
|  | No | REF | REF |  | REF | REF |  |  | REF | REF |  | -- | -- |  |  |
|  | Yes | 0.52 (0.27-1.02) | 0.06 |  | 0.49 (0.25-0.97) | 0.04 |  |  | 1.07 (0.62-1.85) | 0.8 |  | -- | -- |  |  |
|  | Region of maternal birth |  |  | 557 |  |  |  |  |  |  | 899 |  |  |  |  |
|  | Australia or New Zealand | REF | REF |  | REF | REF |  |  | REF | REF |  | -- | -- |  |  |
|  | Asia | 0.37 (0.19-0.74) | <0.01 |  | 0.34 (0.17-0.68) | <0.01 |  |  | 0.57 (0.38-0.85) | <0.01 |  | -- | -- |  |  |
|  | Europe | 1.27 (0.59-2.75) | 0.55 |  | 1.42 (0.62-3.25) | 0.41 |  |  | 1.18 (0.58-2.40) | 0.65 |  | -- | -- |  |  |
|  | Americas | 0.97 (0.26-3.66) | 0.96 |  | 0.77 (0.19-3.20) | 0.72 |  |  | 0.61 (0.13-2.76) | 0.52 |  | -- | -- |  |  |
|  | Africa | 0.26 (0.03-2.52) | 0.24 |  | 0.23 (0.02-2.20) | 0.2 |  |  | 0.52 (0.06-4.28) | 0.54 |  | -- | -- |  |  |
|  | Oceania | 0.00 (0.00-Inf) | 0.98 |  | 0.00 (0.00-Inf) | 0.98 |  |  | 0.57 (0.17-1.98) | 0.38 |  | -- | -- |  |  |
| **Neonatal resuscitation** | BMI category |  |  | 558 |  |  | 546 | 0.59 |  |  | 903 |  |  | 878 | 0.59 |
|  | Normal weight | REF | REF |  | -- | -- |  |  | REF | REF |  | REF | REF |  |  |
|  | Overweight | 0.85 (0.56-1.28) | 0.44 |  | -- | -- |  |  | 1.60 (1.01-2.53) | 0.04 |  | 1.51 (0.95-2.39) | 0.08 |  |  |
|  | Obesity | 1.13 (0.73-1.76) | 0.57 |  | -- | -- |  |  | 1.98 (1.31-2.99) | <0.01 |  | 1.81 (1.19-2.76) | <0.01 |  |  |
|  | Duration of diabetes | 1.03 (1.00-1.06) | 0.06 | 558 | -- | -- |  |  | 1.03 (0.99-1.08) | 0.15 | 903 | -- | -- |  |  |
|  | Age | 0.95 (0.91-0.98) | <0.01 | 558 | 0.94 (0.91-0.97) | <0.01 |  |  | 1.01 (0.98-1.03) | 0.67 | 903 | -- | -- |  |  |
|  | IRSAD score | 1.00 (1.00-1.00) | 0.04 | 547 | -- | -- |  |  | 1.00 (1.00-1.00) | 0.18 | 882 | -- | -- |  |  |
|  | Preexisting hypertension |  |  | 558 |  |  |  |  |  |  | 903 |  |  |  |  |
|  | No | REF | REF |  | -- | -- |  |  | REF | REF |  | REF | REF |  |  |
|  | Yes | 0.87 (0.42-1.79) | 0.71 |  | -- | -- |  |  | 1.78 (1.14-2.77) | 0.01 |  | 1.64 (1.04-2.58) | 0.03 |  |  |
|  | Smoking during pregnancy |  |  | 558 |  |  |  |  |  |  | 903 |  |  |  |  |
|  | No | REF | REF |  | -- | -- |  |  | REF | REF |  | REF | REF |  |  |
|  | Yes | 1.24 (0.64-2.43) | 0.52 |  | -- | -- |  |  | 1.50 (0.95-2.37) | 0.08 |  | 1.47 (0.93-2.33) | 0.1 |  |  |
|  | Region of maternal birth |  |  | 557 |  |  |  |  |  |  | 899 |  |  |  |  |
|  | Australia or New Zealand | REF | REF |  | -- | -- |  |  | REF | REF |  | -- | -- |  |  |
|  | Asia | 0.54 (0.26-1.13) | 0.1 |  | -- | -- |  |  | 0.84 (0.61-1.16) | 0.28 |  | -- | -- |  |  |
|  | Europe | 0.85 (0.39-1.88) | 0.69 |  | -- | -- |  |  | 0.88 (0.44-1.74) | 0.71 |  | -- | -- |  |  |
|  | Americas | 2.02 (0.53-7.66) | 0.3 |  | -- | -- |  |  | 0.66 (0.18-2.39) | 0.52 |  | -- | -- |  |  |
|  | Africa | 0.54 (0.06-5.25) | 0.59 |  | -- | -- |  |  | 1.45 (0.34-6.14) | 0.62 |  | -- | -- |  |  |
|  | Oceania | 0.00 (0.00-Inf) | 0.98 |  | -- | -- |  |  | 1.38 (0.57-3.36) | 0.48 |  | -- | -- |  |  |
| **Neonatal hypoglycaemia** | BMI category |  |  | 554 |  |  | 542 | 0.53 |  |  | 886 |  |  | 861 | 0.61 |
|  | Normal weight | REF | REF |  | -- | -- |  |  | REF | REF |  | REF | REF |  |  |
|  | Overweight | 1.08 (0.71-1.63) | 0.72 |  | -- | -- |  |  | 1.25 (0.80-1.94) | 0.32 |  | 1.15 (0.73-1.80) | 0.55 |  |  |
|  | Obesity | 0.79 (0.51-1.22) | 0.28 |  | -- | -- |  |  | 1.79 (1.21-2.65) | <0.01 |  | 1.53 (1.02-2.30) | 0.04 |  |  |
|  | Duration of diabetes | 1.03 (1.00-1.06) | 0.09 | 554 | 1.03 (0.99-1.06) | 0.11 |  |  | 0.98 (0.94-1.03) | 0.44 | 886 | 0.96 (0.92-1.01) | 0.14 |  |  |
|  | Age | 0.98 (0.95-1.02) | 0.35 | 554 | -- | -- |  |  | 1.04 (1.02-1.07) | <0.01 | 886 | 1.05 (1.02-1.08) | <0.01 |  |  |
|  | IRSAD score | 1.00 (1.00-1.00) | 0.98 | 543 | -- | -- |  |  | 1.00 (1.00-1.00) | 0.3 | 865 | -- | -- |  |  |
|  | Preexisting hypertension |  |  | 554 |  |  |  |  |  |  | 886 |  |  |  |  |
|  | No | REF | REF |  | -- | -- |  |  | REF | REF |  | REF | REF |  |  |
|  | Yes | 0.89 (0.44-1.79) | 0.74 |  | -- | -- |  |  | 2.50 (1.61-3.87) | <0.01 |  | 2.23 (1.40-3.56) | <0.01 |  |  |
|  | Smoking during pregnancy |  |  | 554 |  |  |  |  |  |  | 886 |  |  |  |  |
|  | No | REF | REF |  | -- | -- |  |  | REF | REF |  | REF | REF |  |  |
|  | Yes | 0.91 (0.46-1.79) | 0.78 |  | -- | -- |  |  | 1.62 (1.04-2.53) | 0.03 |  | 1.83 (1.14-2.94) | 0.01 |  |  |
|  | Region of maternal birth |  |  | 553 |  |  |  |  |  |  | 882 |  |  |  |  |
|  | Australia or New Zealand | REF | REF |  | -- | -- |  |  | REF | REF |  | -- | -- |  |  |
|  | Asia | 0.71 (0.37-1.37) | 0.31 |  | -- | -- |  |  | 1.26 (0.92-1.72) | 0.15 |  | -- | -- |  |  |
|  | Europe | 1.29 (0.58-2.91) | 0.53 |  | -- | -- |  |  | 1.58 (0.83-2.99) | 0.16 |  | -- | -- |  |  |
|  | Americas | 0.73 (0.19-2.76) | 0.64 |  | -- | -- |  |  | 1.11 (0.34-3.68) | 0.86 |  | -- | -- |  |  |
|  | Africa | 0.58 (0.08-4.19) | 0.59 |  | -- | -- |  |  | 0.36 (0.04-2.95) | 0.34 |  | -- | -- |  |  |
|  | Oceania | 0.00 (0.00-Inf) | 0.98 |  | -- | -- |  |  | 2.09 (0.88-4.95) | 0.09 |  | -- | -- |  |  |
| **NICU admission** | BMI category |  |  | 228 |  |  | 223 | 0.63 |  |  | 349 |  |  | 337 | 0.63 |
|  | Normal weight | REF | REF |  | -- | -- |  |  | REF | REF |  | -- | -- |  |  |
|  | Overweight | 0.91 (0.49-1.69) | 0.77 |  | -- | -- |  |  | 1.49 (0.71-3.12) | 0.29 |  | -- | -- |  |  |
|  | Obesity | 1.26 (0.61-2.60) | 0.54 |  | -- | -- |  |  | 1.84 (0.97-3.47) | 0.06 |  | -- | -- |  |  |
|  | Duration of diabetes | 1.03 (0.98-1.08) | 0.26 | 228 | -- | -- |  |  | 1.07 (1.00-1.14) | 0.046 | 349 | 1.06 (0.99-1.14) | 0.07 |  |  |
|  | Age | 0.99 (0.95-1.05) | 0.83 | 228 | -- | -- |  |  | 1.07 (1.02-1.11) | <0.01 | 349 | 1.06 (1.02-1.11) | <0.01 |  |  |
|  | IRSAD score | 0.99 (0.99-1.00) | <0.01 | 223 | 0.99 (0.99-1.00) | <0.01 |  |  | 1.00 (0.99-1.00) | 0.13 | 341 | 1.00 (0.99-1.00) | 0.049 |  |  |
|  | Preexisting hypertension |  |  | 228 |  |  |  |  |  |  | 349 |  |  |  |  |
|  | No | REF | REF |  | -- | -- |  |  | REF | REF |  | REF | REF |  |  |
|  | Yes | 0.89 (0.28-2.84) | 0.85 |  | -- | -- |  |  | 2.75 (1.48-5.09) | <0.01 |  | 2.27 (1.19-4.33) | 0.01 |  |  |
|  | Smoking during pregnancy |  |  | 228 |  |  |  |  |  |  | 349 |  |  |  |  |
|  | No | REF | REF |  | -- | -- |  |  | REF | REF |  | -- | -- |  |  |
|  | Yes | 1.13 (0.41-3.16) | 0.81 |  | -- | -- |  |  | 1.24 (0.68-2.27) | 0.49 |  | -- | -- |  |  |
|  | Region of maternal birth |  |  | 228 |  |  |  |  |  |  | 345 |  |  |  |  |
|  | Australia or New Zealand | REF | REF |  | -- | -- |  |  | REF | REF |  | -- | -- |  |  |
|  | Asia | 0.35 (0.13-0.97) | 0.04 |  | -- | -- |  |  | 1.31 (0.79-2.16) | 0.29 |  | -- | -- |  |  |
|  | Europe | 0.76 (0.20-2.79) | 0.67 |  | -- | -- |  |  | 2.38 (0.83-6.85) | 0.11 |  | -- | -- |  |  |
|  | Americas | 0.76 (0.12-4.68) | 0.76 |  | -- | -- |  |  | 3.13 (0.51-19.26) | 0.22 |  | -- | -- |  |  |
|  | Africa | 0.50 (0.03-8.31) | 0.63 |  | -- | -- |  |  | 2.08 (0.13-34.15) | 0.61 |  | -- | -- |  |  |
|  | Oceania | -- | -- |  | -- | -- |  |  | 6.25 (1.22-31.95) | 0.03 |  | -- | -- |  |  |
| **Neonatal hospital LOS > 10 days** | BMI category |  |  | 554 |  |  | 542 | 0.65 |  |  | 886 |  |  | 861 | 0.62 |
|  | Normal weight | REF | REF |  | REF | REF |  |  | REF | REF |  | REF | REF |  |  |
|  | Overweight | 0.68 (0.41-1.13) | 0.13 |  | 0.70 (0.41-1.19) | 0.18 |  |  | 2.55 (1.17-5.53) | 0.02 |  | 2.43 (1.11-5.33) | 0.03 |  |  |
|  | Obesity | 1.39 (0.85-2.26) | 0.18 |  | 1.29 (0.77-2.16) | 0.33 |  |  | 1.87 (0.89-3.94) | 0.1 |  | 1.61 (0.75-3.46) | 0.22 |  |  |
|  | Duration of diabetes | 1.08 (1.04-1.12) | <0.01 | 554 | 1.07 (1.03-1.12) | <0.01 |  |  | 1.06 (1.00-1.13) | 0.06 | 886 | 1.06 (0.99-1.13) | 0.09 |  |  |
|  | Age | 0.99 (0.95-1.03) | 0.58 | 554 | -- | -- |  |  | 1.05 (1.01-1.10) | 0.02 | 886 | 1.04 (1.00-1.09) | 0.06 |  |  |
|  | IRSAD score | 1.00 (0.99-1.00) | 0.03 | 543 | -- | -- |  |  | 1.00 (1.00-1.00) | 0.92 | 865 | -- | -- |  |  |
|  | Preexisting hypertension |  |  | 554 |  |  |  |  |  |  | 886 |  |  |  |  |
|  | No | REF | REF |  | REF | REF |  |  | REF | REF |  | REF | REF |  |  |
|  | Yes | 2.85 (1.41-5.77) | <0.01 |  | 2.52 (1.21-5.27) | 0.01 |  |  | 2.26 (1.23-4.16) | <0.01 |  | 1.87 (0.98-3.58) | 0.06 |  |  |
|  | Smoking during pregnancy |  |  | 554 |  |  |  |  |  |  | 886 |  |  |  |  |
|  | No | REF | REF |  | REF | REF |  |  | REF | REF |  | -- | -- |  |  |
|  | Yes | 2.46 (1.24-4.89) | <0.01 |  | 2.35 (1.16-4.75) | 0.02 |  |  | 1.00 (0.46-2.15) | 1 |  | -- | -- |  |  |
|  | Region of maternal birth |  |  | 553 |  |  |  |  |  |  | 882 |  |  |  |  |
|  | Australia or New Zealand | REF | REF |  | -- | -- |  |  | REF | REF |  | -- | -- |  |  |
|  | Asia | 0.36 (0.12-1.02) | 0.054 |  | -- | -- |  |  | 1.18 (0.71-1.95) | 0.52 |  | -- | -- |  |  |
|  | Europe | 0.51 (0.17-1.50) | 0.22 |  | -- | -- |  |  | 1.41 (0.53-3.78) | 0.49 |  | -- | -- |  |  |
|  | Americas | 1.60 (0.39-6.51) | 0.51 |  | -- | -- |  |  | 0.92 (0.12-7.26) | 0.94 |  | -- | -- |  |  |
|  | Africa | 0.00 (0.00-Inf) | 0.98 |  | -- | -- |  |  | 1.57 (0.19-13.14) | 0.67 |  | -- | -- |  |  |
|  | Oceania | 0.00 (0.00-Inf) | 0.99 |  | -- | -- |  |  | 0.52 (0.07-4.01) | 0.53 |  | -- | -- |  |  |
| **Stillbirth** | BMI category |  |  | 568 |  |  | 556 | -- |  |  | 910 |  |  | 885 | 0.84 |
|  | Normal weight | REF | REF |  | -- | -- |  |  | REF | REF |  | -- | -- |  |  |
|  | Overweight | 0.61 (0.12-3.05) | 0.54 |  | -- | -- |  |  | 0.00 (0.00-Inf) | 0.99 |  | -- | -- |  |  |
|  | Obesity | 0.80 (0.16-4.05) | 0.79 |  | -- | -- |  |  | 2.43 (0.29-20.39) | 0.41 |  | -- | -- |  |  |
|  | Duration of diabetes | 1.00 (0.89-1.12) | 0.95 | 568 | -- | -- |  |  | 1.05 (0.86-1.28) | 0.63 | 910 | -- | -- |  |  |
|  | Age | 0.96 (0.85-1.09) | 0.54 | 568 | -- | -- |  |  | 0.98 (0.85-1.12) | 0.75 | 910 | -- | -- |  |  |
|  | IRSAD score | 1.00 (0.99-1.01) | 0.79 | 557 | -- | -- |  |  | 0.99 (0.98-1.00) | 0.01 | 889 | 0.99 (0.98-1.00) | <0.01 |  |  |
|  | Preexisting hypertension |  |  | 568 |  |  |  |  |  |  | 910 |  |  |  |  |
|  | No | REF | REF |  | -- | -- |  |  | REF | REF |  | REF | REF |  |  |
|  | Yes | 1.66 (0.20-13.54) | 0.64 |  | -- | -- |  |  | 6.45 (1.42-29.34) | 0.02 |  | 7.20 (1.53-33.94) | 0.01 |  |  |
|  | Smoking during pregnancy |  |  | 568 |  |  |  |  |  |  | 910 |  |  |  |  |
|  | No | REF | REF |  | -- | -- |  |  | REF | REF |  | -- | -- |  |  |
|  | Yes | 0.00 (0.00-Inf) | 0.99 |  | -- | -- |  |  | 1.47 (0.17-12.37) | 0.72 |  | -- | -- |  |  |
|  | Region of maternal birth |  |  | 567 |  |  |  |  |  |  | 906 |  |  |  |  |
|  | Australia or New Zealand | REF | REF |  | -- | -- |  |  | REF | REF |  | -- | -- |  |  |
|  | Asia | 0.00 (0.00-Inf) | 0.99 |  | -- | -- |  |  | 0.35 (0.04-3.01) | 0.34 |  | -- | -- |  |  |
|  | Europe | 0.00 (0.00-Inf) | 0.99 |  | -- | -- |  |  | 0.00 (0.00-Inf) | 0.99 |  | -- | -- |  |  |
|  | Americas | 0.00 (0.00-Inf) | 1 |  | -- | -- |  |  | 0.00 (0.00-Inf) | 1 |  | -- | -- |  |  |
|  | Africa | 0.00 (0.00-Inf) | 1 |  | -- | -- |  |  | 0.00 (0.00-Inf) | 1 |  | -- | -- |  |  |
|  | Oceania | 0.00 (0.00-Inf) | 1 |  | -- | -- |  |  | 4.68 (0.52-41.92) | 0.17 |  | -- | -- |  |  |
| Results are presented as odds ratios (OR) with 95% confidence intervals (CI), and p-values. The multivariable models were calculated using stepwise selection based on Akaike Information Criterion (AIC) with both forward and backward selection to determine the optimal set of predictors. -- indicates that the variable was dropped during stepwise selection using AIC. The multivariable models were internally validated using 10-fold cross-validated area under the receiver operating characteristic curve (AUC). BMI classifications include; Normal weight 18.5-25, overweight 25-29.9, obesity 30+.  BMI, body mass index; IRSAD, index of relative socio-economic advantage and disadvantage; SGA, small for gestational age; LGA, large for gestational age; LOS, length of stay; NICU, neonatal intensive care admission. | | | | | | | | | | | | | | | |

**Supplementary table 6: Maternal characteristics of patients with a recorded HbA1c <20 weeks of gestation, by diabetes type**

|  | **Type 1**  N = 104 | **Type 2**  N = 134 | **p-value** |  |
| --- | --- | --- | --- | --- |
| **Age (years), Mean (SD)** | 28.4 (5.2) | 31.4 (6.0) | <0.001^1^ |  |
| **Duration of diabetes (years), Median (IQR)** | 9.0 (1.5, 13.0) | 0.6 (0.0, 3.7) | <0.001^2^ |  |
| **Maternal weight (kg), Median (IQR)** | 68.0 (61.5, 80.0) | 82.5 (72.0, 96.0) | <0.001^2^ |  |
| **Maternal height (cm), Mean (SD)** | 163.9 (6.8) | 164.1 (7.3) | 0.816^1^ |  |
| **BMI (kg/m²), Mean (SD)** | 26.8 (5.1) | 31.6 (6.5) | <0.001^1^ |  |
| **BMI Category (kg/m²), n (%)** |  |  | <0.001^3^ |  |
| Normal weight (BMI 18.5-24.9) | 49 (47.1%) | 23 (17.2%) |  |  |
| Overweight (BMI 25-29.9) | 33 (31.7%) | 29 (21.6%) |  |  |
| Obesity (BMI 30+) | 22 (21.2%) | 82 (61.2%) |  |  |
| **Antenatal care received, n (%)** | 103 (99.0%) | 134 (100.0%) | 0.437^4^ |  |
| **Smoking during pregnancy, n (%)** | 5 (4.8%) | 25 (18.7%) | 0.003^3^ |  |
| **Pre-existing hypertension, n (%)** | <5^5^ | 25 (18.7%) | <0.001^3^ |  |
| **IRSAD Score, Mean (SD)** | 1004.6 (83.8) | 982.2 (70.1) | 0.031^1^ |  |
| Missing (%) | 2 (1.9%) | 2 (1.5%) |  |  |
| **IRSAD Quintile, n (%)** |  |  | 0.004^3^ |  |
| Most disadvantaged quintile | 23 (22.5%) | 30 (22.7%) |  |  |
| 2 | 19 (18.6%) | 48 (36.4%) |  |  |
| 3 | 20 (19.6%) | 18 (13.6%) |  |  |
| 4 | 16 (15.7%) | 24 (18.2%) |  |  |
| Most advantaged quintile | 24 (23.5%) | 12 (9.1%) |  |  |
| Missing (%) | 2 (1.9%) | 2 (1.5%) |  |  |
| **Region of maternal birth, n (%)** |  |  | 0.007^3^ |  |
| Australia and New Zealand | 82 (78.8%) | 77 (57.9%) |  |  |
| Asia | 14 (13.5%) | 45 (33.8%) |  |  |
| Europe | 5 (4.8%) | 6 (4.5%) |  |  |
| Africa | <5^5^ | <5^5^ |  |  |
| Americas | <5^5^ | <5^5^ |  |  |
| Oceania | 0 (0.0%) | <5^5^ |  |  |
| Missing (%) | 0 (0%) | 1 (0.7%) |  |  |
| **Glycaemic control (HbA1c %)** | |  |  |  |
| Pre-Pregnancy, Mean (SD) | | 77 (26) [9.2 (2.4)] | 64 (25) [7.9 (2.4)] | 0.466^1^ |
| Missing (%) | | 89 (86%) | 131 (98%) |  |
| <20 weeks gestation, Median (IQR) | | 52 (44, 62) [6.9 (6.2, 7.8)] | 42 (33, 55) [6.0 (5.2, 7.2)] | <0.001^2^ |
| Trimester 1, Mean (SD) | | 56 (18) [7.3 (1.6)] | 50 (17) [6.6 (1.6)] | 0.006^1^ |
| Missing (%) | | 26 (25%) | 43 (32%) |  |
| Trimester 2, Mean (SD) | | 48 (13) [6.4 (1.1)] | 42 (14) [5.9 (1.2)] | 0.088^1^ |
| Missing (%) | | 73 (70%) | 85 (63%) |  |
| Trimester 3, Mean (SD) | | 49 (12) [6.5 (1.0)] | 44 (6) [6.1 (0.5)] | 0.182^1^ |
| Missing (%) | | 88 (85%) | 126 (94%) |  |
| Post-Pregnancy, Mean (SD) | | 62 (18) [7.7 (1.7)] | 79 (38) [9.3 (3.4)] | 0.264^1^ |
| Missing (%) | | 92 (88%) | 126 (94%) |  |
| ^1^Welch Two Sample t-test  ^2^Wilcoxon rank sum test  ^3^Pearson's Chi-squared test  ^4^Fisher's exact test  ^5^Cell counts less than 5 have been suppressed to maintain confidentiality and comply with ethical guidelines.  SD, standard deviation; IQR, interquartile range; BMI, body mass index; IRSAD, index of relative socio-economic advantage and disadvantage.  HbA1c values are shown as mmol/mol (SD) [% (SD%)] for means or mmol/mol (Q1, Q3) [% (Q1, Q3)] for medians; conversion from % used: mmol/mol = 10.93 × % − 23.5, with SDs and IQRs scaled proportionally. | | |  |  |

**Supplementary table 7: Pregnancy outcomes, by diabetes type in the subset of women with HbA1c available at <20 weeks of gestation**

|  | **Type 1**, N = 568^1^ | **Type 2**, N = 910^1^ | **p-value** |
| --- | --- | --- | --- |
| **Preeclampsia** | 11 (10.6%) | 13 (9.7%) | 0.996^3^ |
| **Gestational hypertension** | 6 (5.8%) | 6 (4.5%) | 0.878^3^ |
| **Postpartum haemorrhage** | 29 (28.2%) | 43 (32.1%) | 0.610^3^ |
| **Normal vaginal delivery** | 11 (10.6%) | 32 (23.9%) | 0.013^3^ |
| **Instrumental delivery** | 14 (13.5%) | 22 (16.4%) | 0.653^3^ |
| **Caesarean section** | 79 (76.0%) | 80 (59.7%) | 0.012^3^ |
| **Maternal hospital LOS (days)** | 5.0 (3.0, 9.0) | 3.0 (2.0, 5.0) | <0.001^4^ |
| **Maternal hospital LOS > 10 days** | 24 (24.0%) | 9 (7.0%) | <0.001^3^ |
| **Maternal death** | 0 (0.0%) | 0 (0.0%) | >0.999^2^ |
| **Gestational age (weeks)** | 37.0 (36.0, 38.0) | 38.0 (37.0, 39.0) | <0.001^4^ |
| **Preterm birth** | 42 (41.2%) | 14 (10.7%) | <0.001^3^ |
| **Birth weight (kg)** | 3.4 (2.8, 3.7) | 3.3 (3.0, 3.6) | 0.128^4^ |
| **SGA** | <5^5^ | 9 (6.9%) | 0.493^3^ |
| **LGA** | 51 (50.0%) | 23 (17.6%) | <0.001^3^ |
| **NICU admission** | 48 (64.9%) | 49 (45.8%) | 0.017^3^ |
| **Neonatal resuscitation** | 34 (33.3%) | 48 (36.6%) | 0.699^3^ |
| **Neonatal hypoglycaemia** | 72 (71.3%) | 57 (44.5%) | <0.001^3^ |
| **Neonatal hospital LOS (days)** | 5.0 (3.0, 9.0) | 3.0 (2.0, 5.0) | <0.001^4^ |
| **Neonatal hospital LOS > 10 days** | 24 (23.8%) | 9 (7.0%) | <0.001^3^ |
| **Stillbirth** | <5^5^ | <5^5^ | >0.999^2^ |
| ^1^n (%), Median (IQR) | | | |
| ^2^Fisher's exact test | | | |
| ^3^Pearson's Chi-squared test | | | |
| ^4^Wilcoxon rank sum test  IQR, interquartile range; SGA, small for gestational age; LGA, large for gestational age; LOS, length of stay; NICU, neonatal intensive care.  ^5^Cell counts less than 5 have been suppressed to maintain confidentiality and comply with ethical guidelines. | | | |

**Supplementary table 8: Odds ratios of adverse pregnancy outcomes in women with type 1 diabetes compared to type 2 diabetes in the subset of women with HbA1c available at <20 weeks of gestation**

|  | **Univariable** | | | **Multivariable** | | |
| --- | --- | --- | --- | --- | --- | --- |
| **Outcome** | **OR (95% CI)** | **p-value** | **N** | **OR (95% CI)** | **p-value** | **N** |
| **Preeclampsia** | 1.10 (0.47-2.58) | 0.82 | 238 | 2.70 (0.85-8.53) | 0.09 | 233 |
| **Gestational hypertension** | 1.31 (0.41-4.20) | 0.65 | 238 | -- | -- | 233 |
| **Postpartum haemorrhage** | 0.83 (0.47-1.46) | 0.51 | 237 | -- | -- | 232 |
| **Caesarean section** | 2.13 (1.21-3.77) | <0.01 | 238 | 3.11 (1.49-6.52) | <0.01 | 233 |
| **Maternal hospital LOS > 10 days** | 4.18 (1.83-9.51) | <0.01 | 228 | 2.95 (0.93-9.30) | 0.06 | 223 |
| **Preterm birth** | 5.85 (2.95-11.59) | <0.01 | 233 | 4.10 (1.72-9.80) | <0.01 | 228 |
| **SGA** | 0.55 (0.16-1.86) | 0.34 | 233 | -- | -- | 228 |
| **LGA** | 4.70 (2.58-8.53) | <0.01 | 233 | 5.01 (2.59-9.69) | <0.01 | 228 |
| **Neonatal resuscitation** | 0.86 (0.50-1.49) | 0.6 | 233 | 0.49 (0.25-0.98) | 0.04 | 228 |
| **Neonatal hypoglycaemia** | 3.09 (1.77-5.40) | <0.01 | 229 | 3.08 (1.68-5.63) | <0.01 | 224 |
| **NICU admission** | 2.19 (1.18-4.04) | 0.01 | 181 | 2.08 (1.04-4.14) | 0.04 | 176 |
| **Neonatal hospital LOS > 10 days** | 4.12 (1.81-9.38) | <0.01 | 229 | 2.94 (0.93-9.30) | 0.07 | 224 |
| **Stillbirth** | 0.86 (0.14-5.27) | 0.87 | 238 | -- | -- | 233 |
| Results are presented as odds ratios (OR) with 95% confidence intervals (CI) and p-values. If no OR is reported within the multivariable analysis, then diabetes type was excluded during the stepwise multivariable model selection based on Akaike Information Criterion (AIC). See supplementary table 3 for the full models with the included covariates.  SGA, small for gestational age; LGA, large for gestational age; LOS, length of stay; NICU, neonatal intensive care. | | | | | | |

**Supplementary table 9: Odds ratios of adverse pregnancy outcomes in women with obesity compared to normal weight in either type 1 or type 2 diabetes in subset of women with HbA1c available at <20 weeks gestation.**

|  | **Type 1** | | | | | | **Type 2** | | | | | |
| --- | --- | --- | --- | --- | --- | --- | --- | --- | --- | --- | --- | --- |
|  | **Univariate** | | | **Multivariate** | | | **Univariate** | | | **Multivariate** | | |
| **Outcome** | **OR (95% CI)** | **p-value** | **N** | **OR (95% CI)** | **p-value** | **N** | **OR (95% CI)** | **p-value** | **N** | **OR (95% CI)** | **p-value** | **N** |
| **Preeclampsia** | 2.42 (0.44-13.35) | 0.3 | 104 | -- | -- | 102 | 2.71 (0.32-23.05) | 0.36 | 134 | -- | -- | 131 |
| **Gestational hypertension** | 1.12 (0.19-6.80) | 0.9 | 104 | 1.10 (0.18-6.65) | 0.92 | 102 | 2.04E7 (0.00-Inf) | 0.99 | 134 | -- | -- | 131 |
| **Postpartum haemorrhage** | 0.26 (0.07-1.03) | 0.053 | 103 | 0.22 (0.06-0.91) | 0.03 | 101 | 1.55 (0.55-4.41) | 0.41 | 134 | -- | -- | 131 |
| **Caesarean section** | 1.30 (0.36-4.73) | 0.68 | 104 | -- | -- | 102 | 1.70 (0.67-4.36) | 0.26 | 134 | -- | -- | 131 |
| **Maternal hospital LOS > 10 days** | 3.50 (1.01-12.14) | 0.046 | 100 | -- | -- | 98 | 1.44 (0.16-13.28) | 0.75 | 128 | 0.46 (0.01-14.08) | 0.65 | 125 |
| **Preterm birth (<37 weeks)** | 4.15 (1.37-12.61) | 0.01 | 102 | 3.07 (0.91-10.33) | 0.07 | 100 | 0.54 (0.14-1.99) | 0.35 | 131 | -- | -- | 128 |
| **SGA** | 0.00 (0.00-Inf) | 0.99 | 102 | -- | -- | 100 | 1.49 (0.16-13.68) | 0.72 | 131 | -- | -- | 128 |
| **LGA** | 1.93 (0.65-5.75) | 0.23 | 102 | -- | -- | 100 | 2.11 (0.56-7.99) | 0.27 | 131 | 1.96 (0.51-7.57) | 0.32 | 128 |
| **Neonatal resuscitation** | 1.05 (0.36-3.09) | 0.92 | 102 | -- | -- | 100 | 1.09 (0.41-2.90) | 0.87 | 131 | -- | -- | 128 |
| **Neonatal hypoglycaemia** | 3.29 (0.83-13.01) | 0.09 | 101 | -- | -- | 99 | 1.44 (0.55-3.80) | 0.45 | 128 | -- | -- | 125 |
| **NICU admission** | 4.19 (0.79-22.25) | 0.09 | 74 | -- | -- | 72 | 0.77 (0.27-2.17) | 0.62 | 107 | -- | -- | 104 |
| **Neonatal hospital LOS > 10 days** | 3.23 (0.94-11.09) | 0.06 | 101 | -- | -- | 99 | 1.44 (0.16-13.28) | 0.75 | 128 | 0.46 (0.01-14.08) | 0.65 | 125 |
| **Stillbirth** | 2.32E8 (0.00-Inf) | 1 | 104 | 2.32E8 (0.00-Inf) | 1 | 102 | 3.25E7 (0.00-Inf) | 1 | 134 | -- | -- | 131 |
| Results are presented as odds ratios (OR) with 95% confidence intervals (CI), and p-values. If no OR is reported within the multivariate analysis, then BMI category was excluded during the stepwise multivariate model selection based on Akaike Information Criterion (AIC). See supplementary material for full models.  SGA, small for gestational age (<10th percentile); LGA, large for gestational age (>90th percentile); LOS, length of stay; NICU, neonatal intensive care. | | | | | | | | | | | | |
